# Suppressing high-dimensional crystallographic defects for ultra-scaled DNA arrays

**DOI:** 10.1038/s41467-022-30441-1

**Published:** 2022-05-16

**Authors:** Yahong Chen, Chaoyong Yang, Zhi Zhu, Wei Sun

**Affiliations:** 1 Collaborative Innovation Center of Chemistry for Energy Materials, The MOE Key Laboratory of Spectrochemical Analysis and Instrumentation, State Key Laboratory of Physical Chemistry of Solid Surfaces, Key Laboratory of Chemical Biology of Fujian Province, Department of Chemical Biology, College of Chemistry and Chemical Engineering, Xiamen University, Xiamen, 361005 China; 2 Key Laboratory for the Physics and Chemistry of Nanodevices and Center for Carbon-Based Electronics, School of Electronics, Peking University, Beijing, 100871 China; 3 Institute of Molecular Medicine, Renji Hospital, School of Medicine, Shanghai Jiao Tong University, Shanghai, 200127 China

**Keywords:** DNA nanostructures, Organizing materials with DNA, Nanoscale devices

## Abstract

While DNA-directed nano-fabrication enables the high-resolution patterning for conventional electronic materials and devices, the intrinsic self-assembly defects of DNA structures present challenges for further scaling into sub-1 nm technology nodes. The high-dimensional crystallographic defects, including line dislocations and grain boundaries, typically lead to the pattern defects of the DNA lattices. Using periodic line arrays as model systems, we discover that the sequence periodicity mainly determines the formation of line defects, and the defect rate reaches 74% at 8.2-nm line pitch. To suppress high-dimensional defects rate, we develop an effective approach by assigning the orthogonal sequence sets into neighboring unit cells, reducing line defect rate by two orders of magnitude at 7.5-nm line pitch. We further demonstrate densely aligned metal nano-line arrays by depositing metal layers onto the assembled DNA templates. The ultra-scaled critical pitches in the defect-free DNA arrays may further promote the dimension-dependent properties of DNA-templated materials.

## Abstract (web summary)

DNA nanofabrication techniques have huge potential for the patterning of electronic materials and devices but suffer from defects which become more significant at lower scales. Here, the authors report on a study into the causes of line defects and develop criteria for reducing defects demonstrating this technique.

## Introduction

Continuously scaling of modern electronics are built on the synthetic capability of forming densely-aligned defect-free polymeric patterns^1,2^. For instance, three-dimensional (3D) space-and-line pattern, written on the polymeric photoresists^3^, has been used for fabricating diverse electronic components, including semiconductor channels and metal contacts with prescribed pitch (center-to-center spacing between two neighboring features) and line widths. Towards the projected sub-1 nm technology node of the ultra-scaled electronics and future quantum devices, a critical line pitch shall be scaled down to sub-10 nm^4,5^. However, defects become more dominated with the line pitch scaling down. For instance, the microbridges between lines are produced in photolithography because the limited resolution cannot differentiate different lines^6,7^. Meanwhile, in the directed self-assembly of copolymer, the entropy-driven dislocated lines emerge, and are difficult to be fully removed via simple thermal annealing due to the kinetic barriers^8–11^.

Using self-assembled 3D DNA patterns as structural templates, the emerging DNA-directed nano-fabrication provides a high-resolution scaling framework for diverse materials and devices^12–23^. DNA self-assembly encodes the designer device architecture information into the connectivity and the complex sequences of the composing single-stranded DNA building blocks^24–35^. Programmable DNA templating patterns are thus in silico designed and scalable assembled via controllable dynamic assembly pathways^36–38^. Further transferring the pattern information from the 3D DNA templates into the electronic materials, such as carbon nanotubes^39–42^ and Si^38^, constructs the prescribed device architectures with both sub-20 nm uniform pitch^38,40,42^ and high device performance^43^. Although individual DNA pattern may be of micrometer in length, it could be further aligned into larger-scale ordered arrays via hierarchical surface assembly^43–45^.

Towards the sub-1 nm technology node, scaling DNA-directed nano-fabrication requires the correct assembly of periodic defect-free DNA lines with sub-10 nm line pitches. In self-assembled DNA patterns, the intrinsic physical properties of DNA helix (i.e., chirality, base number per turn, and crossover density) may induce lattice twisting^27^, which can be corrected via corrugated designs^13,46–49^ and optimized basepair number per helical turn^50^. Meanwhile, the incorporation errors of individual DNAs (i.e., 0D defects, such as missing, duplicating, and local swinging of individual DNA building block)^51^ are occurring at a rate of 0.3–20%^33,52–54^, as results of the overall contributions from different designs^55,56^, synthetic quality^57^, concentrations of DNA motifs^33,38^, the assembly conditions^52,58^, and the measurement accuracy^52–54^, besides the impact of DNA sequence^55,58^. However, because 3D periodic DNA lines typically consist of thousands of DNA building blocks^33,59^, individual 0D incorporation errors of single DNA do not affect the approximate shape of the DNA lines and may not inevitably lead to the pattern failure. Instead, pattern failures arise when the simultaneous accumulations of tens to hundreds of mis-assembled DNAs within nearby lattices extend into higher dimensions^56,59,60^, including 1D dislocated lines and 2D domains of distinct lattice orientations. Even though these high-dimensional defects correlate with the majority of pattern failures^33,38,56,59^, there still lack an effective strategy to suppress them in the periodic DNA patterns. Furthermore, the defective DNA patterns exhibit similar molecular weights and dimensions to those of the correctly assembled ones, presenting challenges for effective purification via conventional separation methods^33,61^.

Here, using micron-scale DNA line arrays (corresponding to the space-and-line pattern in photoresists) as model systems, we explore the key parameters determining the formation of high-dimensional crystallographic defects during the pitch scaling. We first assemble line arrays with programmable *x*-direction pitches ranging from 8.2 nm to 15.3 nm. We find that, starting from locally swinging DNA bricks, the lattice penetration into neighboring unit cells produces the high-dimensional crystallographic defects. And the lattice penetration dynamics decreases at larger sequence periodicity. As results, the line defect rate (LDR) is inversely proportional to the *x*-direction sequence periodicity, and independent of the widths or specific sequences of individual lines. At 8.2-nm line pitch, the LDR value is higher than 70%, among which 73% is 1D line dislocation and 27% is 2D grain boundaries. In contrast, LDR at 15.3-nm line pitch is largely decreased to 1.7%, and all of the defects are 2D grain boundaries. In a defective pattern, the 1D line dislocations appear during the early-stage pattern formation and locate in the central regions of the pattern; whereas 2D grain boundaries appear at latter stages of the pattern formation around the pattern peripherals. To suppress the defects during pitch scaling, we assign orthogonal sequence sets (OSE) for neighboring repeating unit cells. Thus, for a 7.5-nm line pitch, the repeating unit cells of identical sequences are separated at a periodicity of 15 nm. Using this approach, we are able to lower the LDR (less than 1% with OSE) by nearly two orders of magnitude compared to those at similar line pitch but without OSE (74%). Finally, as a proof-of-concept demonstration for the DNA-directed nano-fabrication, we scale the critical pitches of the nickel (Ni) and palladium (Pd) line arrays using the defect-free DNA templates.

## Results

### Design and assembly of DNA pattern

**Fig. 1:**
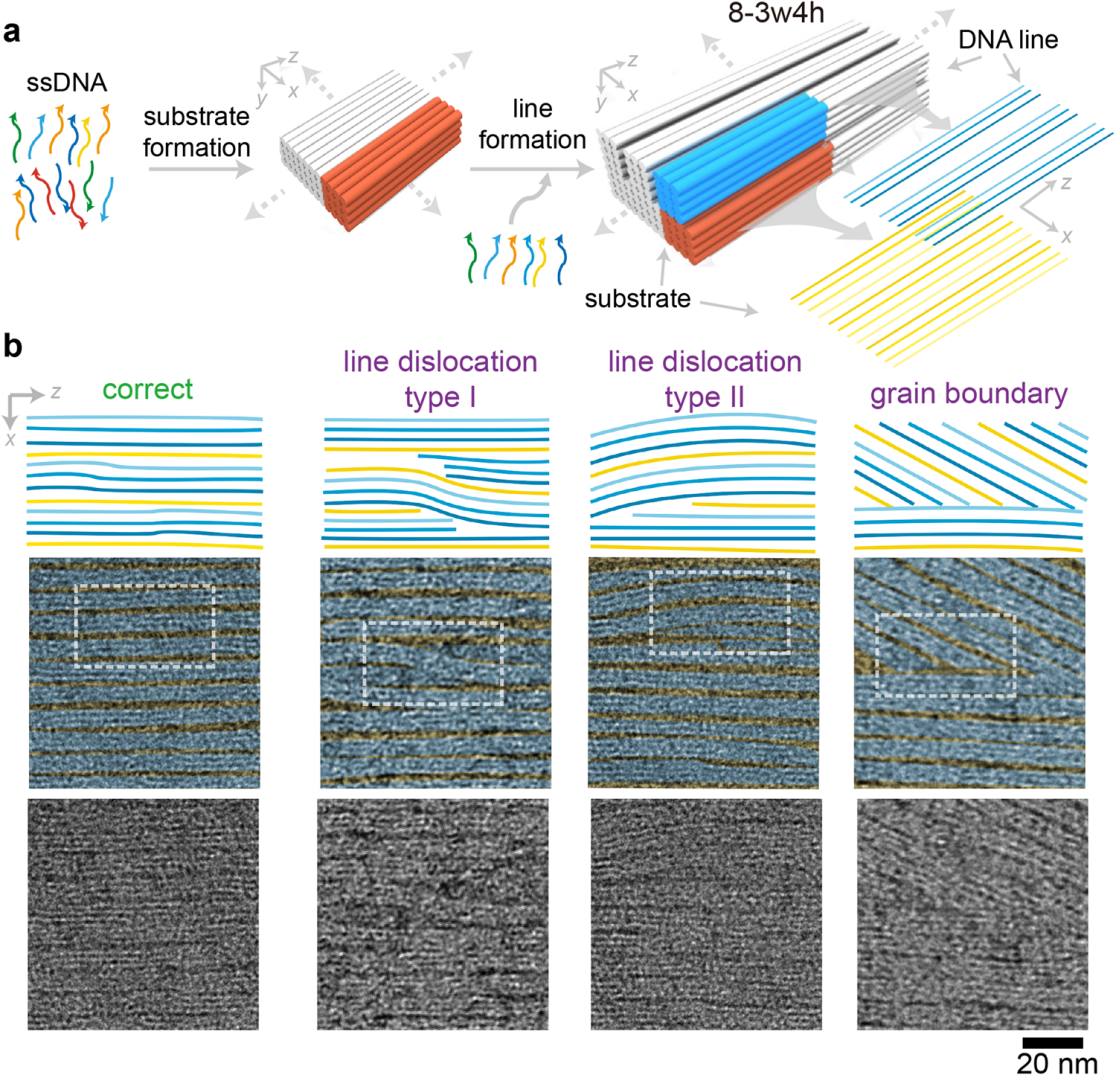
High-dimensional crystallographic defects of 8-3w4h. **a** Assembly pathway for the DNA line arrays. The orange and the blue bundles represent the substrate and the line modules in one repeating unit cell, respectively. The dashed arrows indicate the lattice extension directions. The gradient colored lines correspond to the helical orientations of the DNA lattices. Specifically, yellow gradient colors denote the lattice orientations of the heterogeneous sequenced substrate module; blue gradient colors denote the lattice orientations of the heterogeneous sequenced line module. In 8-3w4h, 8 denotes the line pitch along the *x* direction, 3w denotes the 3 layers of helices for DNA line along the *x* direction, and 4h denotes the 4 layers of helices for DNA line along the *y* direction. **b** Schematics and TEM images for the representative assembly morphologies at 8.2 nm line pitch along the *x* direction. From left to right, the morphologies of the correctly assembled lines, type I line dislocation, type II line dislocation, and grain boundary. Each lane corresponds to one specific assembly morphology. Top, line schematics along the *x*-*z* projection plane. Middle, fake-colored TEM images. Yellow and blue colors denote the space and line regions, respectively. White dashed boxes indicate the schematic area in the top row. Bottom, raw TEM images. The scale bar is 20 nm.

We first explored the defect formation in 8.2-nm pitch DNA line arrays. The DNA patterns were designed using DNA brick crystal approach^59^. In the repeating unit cell of the DNA template, we designed two structural modules, i.e., DNA substrate in the bottom and DNA line on the top (Fig. 1a, namely 8-3w4h, where 8 denoted the line pitch along the *x* direction, 3w denoted the 3 layers of helices for DNA line along the *x* direction, and 4h denoted the 4 layers of helices for DNA line along the *y* direction). DNA substrate and DNA line modules composed of 4 helices × 4 helices × 94 basepairs and 3 helices × 4 helices × 94 basepairs along the *x-y*-*z* directions, producing designed line pitch and width values of 8.4 nm and 6.3 nm (calculated from 2.1-nm diameter per dehydrated helix) along *x* direction, respectively. Extending the repeating unit cells along the *x*-*z* directions yielded the designer arrays of 3D DNA lines. Notably, the line pitch and the sequence periodicity along *x* direction were designed to be identical to the width of substrate module.

We used the modular epitaxy approach to assemble the designed DNA patterns (Fig. 1a)^38^. A 12 µL mixture of unpurified DNA bricks from the DNA substrate module (pH 7.9, containing 800 nM of each DNA brick, 5 mM Tris, 1 mM EDTA, and 40 mM MgCl_2_ in the solution, without careful adjustment of each DNA brick stoichiometry) was sequentially incubated at 85 °C for 15 min, 44 °C for 12 h, 39 °C for 24 h, and 31 °C for 8 h, for effective nucleation and substrate formation. Next, a 59 µL mixture of un-purified DNA bricks (pH 7.9, containing 400 nM of each DNA brick, 5 mM Tris, 1 mM EDTA, and 40 mM MgCl_2_ in the solution, without careful adjustment of each DNA brick stoichiometry) from both the DNA substrate and the DNA line modules was mixed with the pre-annealed solution, and incubated sequentially at 39 °C for 48 h, and 33 °C for 48 h. During this time, newly added DNA bricks were assembled onto the growing substrates, forming the prescribed DNA line arrays patterns. After assembly, the crude product (without any purification) was deposited onto the glow-discharged copper grid, and negatively stained for imaging with a transmission electron microscope (TEM).

### Crystallographic defects at 8.2-nm line pitch

Under TEM imaging, individual assembled pattern displayed a leaf-like morphology (Supplementary Fig. 1). The assembled patterns exhibited wide distributions of lengths and widths, ranging from 0.5 µm to 2 µm in length along *z* direction, and 100 nm to 300 nm in width along *x* direction. Parallel defect-free DNA line arrays displayed the alternative narrow dark (i.e., space, marked with yellow color) and wide bright (i.e., line, marked with blue color) morphologies in the zoomed-in TEM images (Fig. 1b, left). Measured line pitch along *x* direction was 8.2 ± 0.4 nm, with the line width of 6.4 nm ± 0.5 nm (more than 100 DNA lines were counted, Supplementary Fig. 11 and Table 1). Slight differences between the measured values and the designed values of the line pitch and the line width were ascribed to the statistics variation of small counted sample size.

Besides the prescribed parallel DNA lines, we also observed line dislocations (Fig. 1b, middle) and grain boundaries (Fig. 1b, right), which were signatures of typical high-dimensional crystallographic defects (more TEM images in Supplementary Fig. 1). Line dislocation referred to parallel DNA lines penetrating into neighboring line lattices, and all the DNA lines remained parallel except at the defect regions. Depending on if the total line numbers changed, the line dislocations were categorized into type I (unchanged) or type II (changed).

For the type I line dislocation (Fig. 1b, middle left), although the dislocated DNA lines remained continuous, other parallel DNA lines encountering the boundary of the line dislocations stopped growth at the defect regions (white box area in Fig. 1b, middle left); whereas outside the defect regions, all the DNA lines resumed extension along the *z* direction. For type II line dislocation (Fig. 1b, middle right), several layers of DNA lattices (right part of the white box area), from both substrate and line modules, were depleted at the defect regions (left part of the white box area), leading to smaller line numbers and permanently disappeared lines at the left side of the defect region (Fig. 1b, middle right). Notably, the crystallinity of the DNA helices decreased at the defect regions, displaying partially formed unclear lattices.

In the cases where multiple defective DNA lines were not parallel to the correctly assembled DNA lines, grain boundaries appeared between domains of different line orientations (Fig. 1b, right). Within each domain, the DNA lines remained parallel; whereas at the grain boundaries, defective DNA lines either stopped growth or merged into other continuous DNA lines. Notably, line dislocations located in the central regions of a defective DNA pattern. In contrast, grain boundaries mainly located at the peripheral regions.

**Fig. 2:**
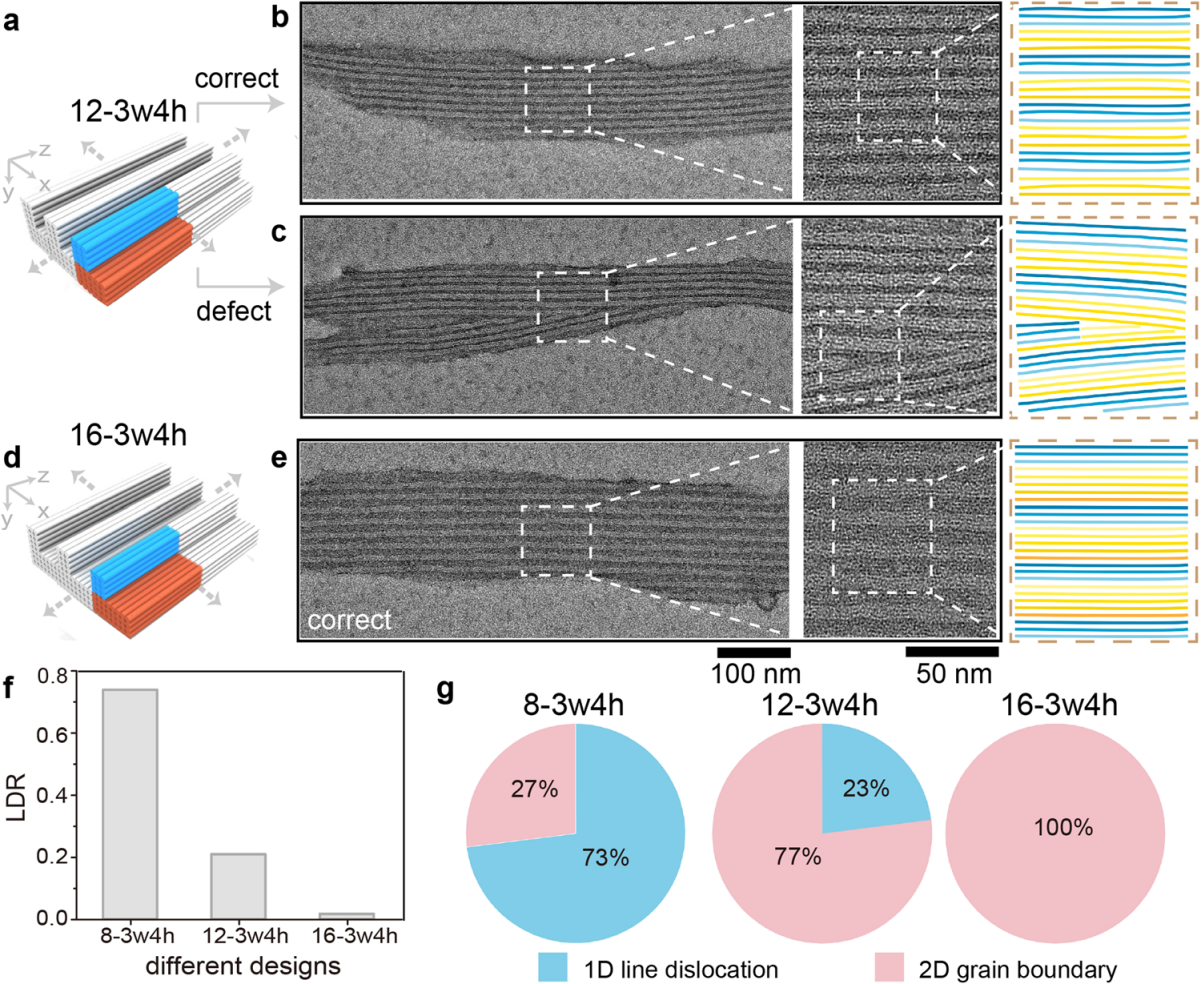
Crystallographic defects at different line pitches. Design schematic (**a**) and assembly morphologies (correctly assembled lines in **b**, pattern defects in **c**) for the representative DNA line arrays (12-3w4h) with 11.5-nm line pitch along *x* direction. Design schematic (**d**) and assembly morphology (correctly assembled lines in **e**) for the representative DNA line arrays (16-3w4h) with 15.3-nm line pitch along *x* direction. Because the pattern defects are rare at 15.3-nm pitches, they are not considered as the representative assembly morphologies. In **b**, **c**, and **e**, zoomed-out (left), zoomed-in (center) TEM images, and the line schematics (right) along the *x-z* projection plane. The scale bars are 100 nm (left) and 50 nm (center). LDR values (**f**) and the compositions of the high-dimensional pattern defects (**g**) for DNA line arrays of 8-3w4h, 12-3w4h, and 16-3w4h. The ratios between 1D line dislocation and 2D grain boundary in **g** are counted from more than 30 independent defective DNA brick crystals in each design. Enlarging the line pitches changes the compositions of the high-dimensional pattern defects, from 1D line dislocation as the major defect (73%) to the dominating 2D grain boundary (i.e., 100%).

We defined the LDR as the total number of DNA patterns with at least one high-dimensional pattern defect divided by the total number of the counted DNA patterns. For 8-3w4h, the LDR was around 74% according to TEM counting (42 individual patterns were counted, Fig. 2f). Individual DNA pattern may contain multiple pattern defects, and 34% of the total DNA lines (counted from defective patterns only) were defective. Among all the pattern defects, more than 70% originated from the 1D line dislocations, and the other portions were the 2D grains of different line orientations (Fig. 2g).

The crossover angles, defined as the orientation difference between a defective line and the correctly assembled parallel lines, were less than 20° in all the counted line dislocations and grain boundaries. The small crossover angles indicated that some composing DNA bricks could swing away from the ideal helical direction and extend the defective lattices thereafter, even though fully-hybridized DNA bricks were designed to be rigid and extending along the helical direction (with an ideal crossover angle of 0°). Such local swing freedom of individual DNAs could originate from the mis-synthesized oligonucleotides, floppy single-stranded overhangs of partially hybridized DNA bricks, or the isomerization of double-stranded DNA (dsDNA) bricks^62^. In addition, within the defect regions, we kept 10.45 basepairs per helical turn, identical to that in the correctly assembled parallel DNA lines. Therefore, the local swing of DNA bricks was not induced by the stress of inserted or depleted nucleotides.

### Correlation between crystallographic defects and line pitch

By programming the widths of the repeating unit cells, we demonstrated DNA lines with larger line pitches along *x* direction (Fig. 2). Within each pattern, we kept identical DNA line module on top (i.e., 3 helices × 4 helices × 94 basepairs along the *x-y-z* directions, Fig. 2a, d). In the bottom DNA substrates modules, 6 helices × 4 helices × 94 basepairs, and 8 helices × 4 helices × 94 basepairs were used for the designed line pitches of 12.6 nm and 16.8 nm (namely 12-3w4h and 16-3w4h, respectively, calculated from 2.1 nm diameter per dehydrated DNA helix), respectively.

Using identical assembly approach to that of 8-3w4h, we assembled the designer DNA lines with larger pitches. Both the leaf-like morphologies and the pattern dimension distributions were similar to those of 8-3w4h (Supplementary Figs. 2 and 3). For the correctly assembled patterns (Fig. 2b, e), the line pitches were measured as 11.5 nm ± 0.4 nm in 12-3w4h and 15.3 nm ± 0.7 nm in 16-3w4h (100–200 DNA lines were counted, Supplementary Fig. 11 and Table 1) along *x* direction. While the line widths were similar (6.6 nm ± 0.2 nm in 12-3w4h and 6.9 nm ± 0.3 nm in 16-3w4h), the spacing values between neighboring lines were increased from 4.9 nm in 12-3w4h to 8.4 nm in 16-3w4h.

The formation of pattern defects (Fig. 2c, more TEM images in Supplementary Figs. 2 and 3) was dependent on the line pitch. When the line pitch increased, the LDR decreased significantly (Fig. 2f). For 12-3w4h, the LDR was 21% (counted from 43 individual patterns), around 28% of that at 8.2-nm line pitch; whereas for 16-3w4h, the LDR was around 1.7% (counted from 112 individual patterns), around 2% of that at 8.2-nm line pitch. Within a defective 12-3w4h pattern, around 34% DNA lines were defective, similar to that of 8-3w4h. But for a defective 16-3w4h pattern, less than 7% DNA lines were defective. Meanwhile, the compositions of the high-dimensional crystallographic defects varied at different line pitches. The ratio between 1D line dislocations and 2D grain boundaries decreased from 23%:77% for 12-3w4h to 0%:100% for 16-3w4h (Fig. 2g, Supplementary Fig. 10).

Even with similar local swing freedom of individual DNA bricks (the crossover angles less than 20°), the overall LDR values (1D line dislocations and 2D grain boundaries combined) of the assembled DNA patterns increased by more than 40 folds simply by decreasing the sequencing periodicity from 15.3 nm to 8.2 nm. Therefore, the inverse correlation between with the sequence periodicity and the LDR value correlated mainly with the defective lattice penetration, rather than differences in the local swinging of individual DNA bricks. We further explored other parameters affecting the defective lattice penetration, including different line designs and the assembly dynamics.

### Effects of different line designs to crystallographic defects

**Fig. 3:**
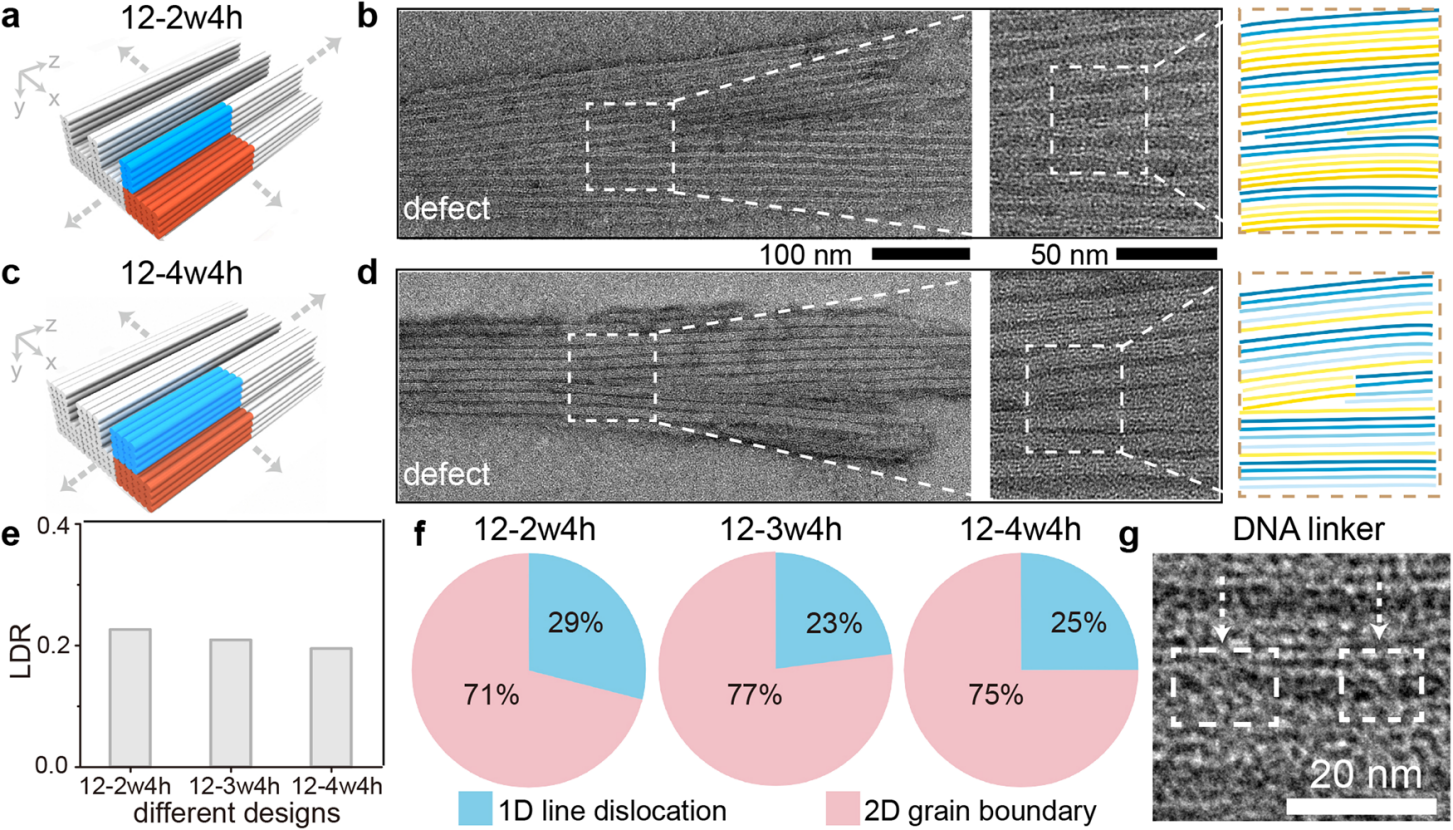
Crystallographic defects for different line designs. Design schematic (**a** and **c**), zoomed-out (left), zoomed-in (center) TEM images, and the line schematics (right) along the *x*–*z* projection plane (**b** and **d**) for the representative pattern defects of DNA line arrays (12-2w4h in **a** and **b** and 12-4w4h in **c** and **d**). The scale bars are 100 nm (left) and 50 nm (center). LDR values (**e**) and the compositions of the high-dimensional crystallographic defects (**f**) for DNA lines arrays of 12-2w4h, 12-3w4h, and 12-4w4h. **g** Intermediate DNA linkers (indicated by the white arrows) between parallel DNA lines, indicating defect penetration is blocked by correct lattices nearby. The scale bar is 20 nm.

**Fig. 4:**
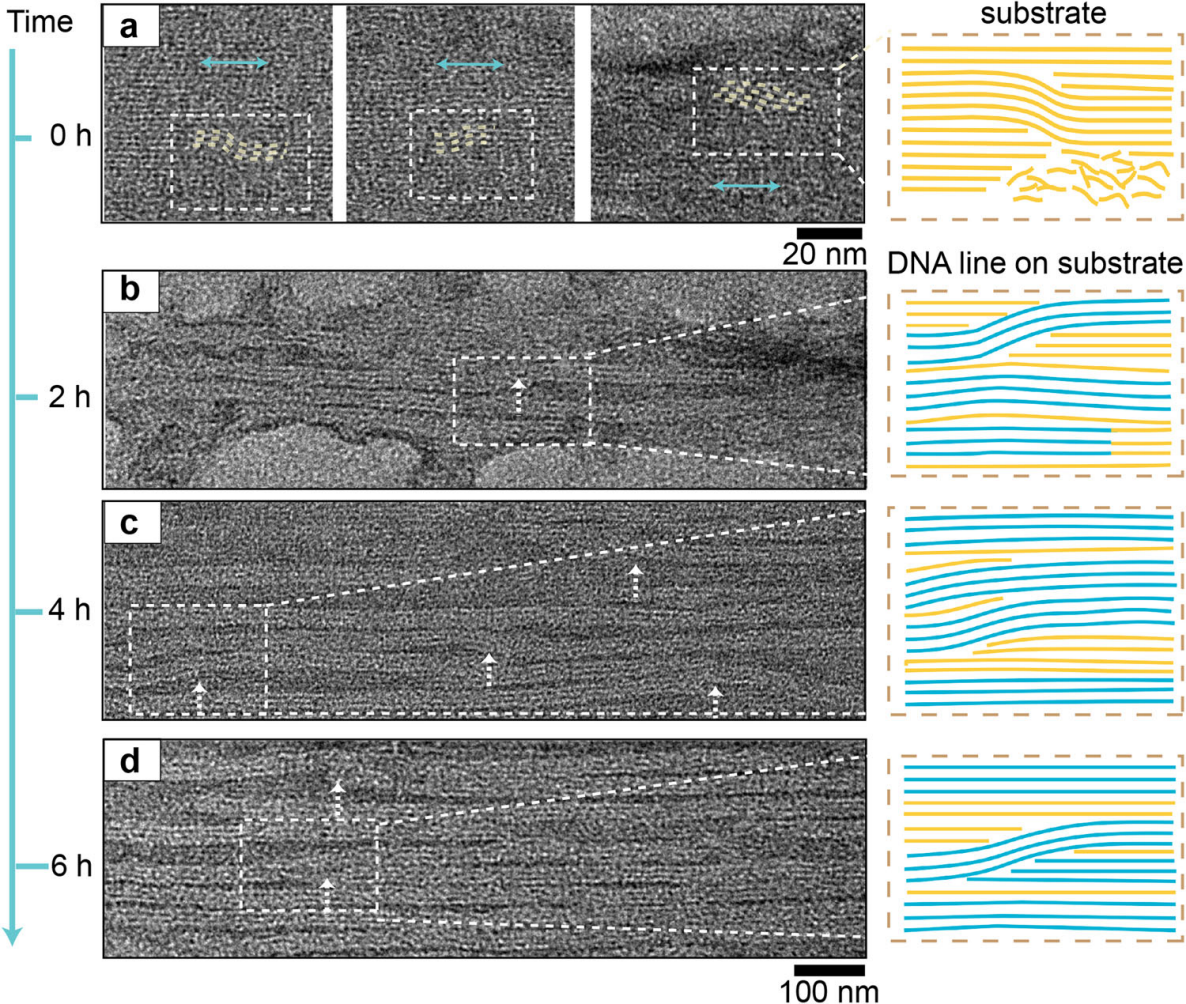
Structural evolving for high-dimensional crystallographic defects. TEM images (left) and the line schematics along the *x*–*z* projection plane for the representative pattern morphologies (right) after substrate formation (*t* = 0, **a**) and line assembly (*t* = 2 h, 4 h, and 6 h, **b**–**d**). The scale bars are 20 nm in **a** and 100 nm in **b**–**d**. In **a**, white dashed boxes denote the typical pattern defect regions, and the line dislocations in the substrate are marked with yellow dashed lines. The cyan arrows indicate the helical directions of the correct DNA lattices. From **b** to **d**, the white arrows indicate typical line dislocations. The presence of typical line dislocations blocks the correct lattice extension of the neighboring DNA lines.

We next verified the effect of different line designs to the defect formation. We used identical substrate module (i.e., 6 helices × 4 helices × 94 basepairs along the *x*-*y*-*z* directions) for a constant designed line pitch of 12.6 nm (calculated from 2.1 nm diameter per dehydrated DNA helix). Meanwhile, we altered the designed line widths from 4.2 nm to 8.4 nm by changing the line modules from 2 helices × 4 helices × 94 basepairs (namely 12-2w4h, Fig. 3a) and 4 helices × 4 helices × 94 basepairs (namely 12-4w4h, Fig. 3c) along the *x*-*y*-*z* directions, respectively. All the DNA line modules were designed with heterogeneous sequences.

Different line designs and sequences sets did not significantly affect the crystallographic defects (Fig. 3b, d, Supplementary Figs. 4, 5 and 12, Table 1). For example, the LDR values were 22% (62 individual patterns were counted), 21%, and 19% (41 individual patterns were counted) for 12-2w4h, 12-3w4h, and 12-4w4h (Fig. 3e), respectively. Other pattern parameters, including the defect morphologies, the ratio between the line dislocations and the grain boundaries (Fig. 3f), and the crossover angles, were also similar regardless of line designs.

The deterministic effect of the *x*-direction line pitch (also the sequence periodicity along *x* direction) to the pattern defects also applied to different pitch values. For example, using identical 8.4-nm designed substrate width (i.e., substrate module of 4 helices × 4 helices × 94 basepairs along the *x-y*-*z* directions in Fig. 1a), scaling the line spacing to zero (namely 8-4w4h, Supplementary Figs. 6 and 7) still preserved similar crystallographic defect morphologies and LDR value (71%, 42 individual patterns were counted) to those with 2-nm line spacing (i.e., the design of 8-3w4h in Fig. 1a, with 74% LDR value).

Notably, in 12-4w4h, we occasionally observed that parallel DNA lines were linked side-by-side via partially formed small lattices, i.e., single to triple-layer DNA helices (Fig. 3g). These DNA linkers displayed larger angle deviation from neighboring parallel DNA lines (up to 60°), but much smaller dimensions (typically 2 nm to 6 nm in width and 4 nm to 6 nm in length, similar length to that of single dsDNA brick) compared to typical line dislocations (Fig. 3b, d, 8 nm in width). And the DNA linkers, composed of a few defective DNA bricks, stopped penetrating at the boundary of the intact neighboring lattices; as results, they did not further form the line dislocations. Considering large distorted orientations and incomplete lattices structures, DNA linkers appeared to be the intermediate states between the correctly assembled DNA lines and typical line defects, where only few defective DNA bricks were confined locally. Meanwhile, both the orientation and the shape integrity of the parallel DNA lines were not affected by the DNA linkers. Therefore, the presence of DNA linkers was not counted as line defects.

The competing between the defective lattices penetrating and the correct assembly of neighboring lattices thus determined the effectiveness of defect formation. When the penetration of the defective lattices occurred prior to the correct lattice assembly, subsequent assembly of correct lattices were blocked by defective lattices, producing high-dimensional crystallographic defects. In contrast, if intact neighboring lattices first assembled, successive extensions of defective lattices would be eliminated, leaving either intermediate DNA linkers as in Fig. 3g or few 0D point defects.

### Structural evolving at different assembly durations

Using 8-3w4h as model system, we further analyzed the defect formation at different growth times (0–6 h after substrate formation). After introducing the composing DNA bricks of the line modules into the pre-assembled substrate modules, we quickly pipetted out few microliter solutions at three selected time durations (2 h, 4 h, and 6 h), and cooled them at 4 °C to freeze the assembly process. The crude products were directly imaged by TEM without any purification.

Directly after substrate formation (*t* = 0), the assembled DNA patterns exhibited flat surface morphologies as designed (Fig. 4a, Supplementary Fig. 14). Meanwhile, zoomed-in TEM images revealed the appearance of high-frequency irregular lattice mismatches and line dislocations within the assembled substrates (yellow lines in Fig. 4a denoted several representative crystallographic defects within the substrates), indicative of defect formation at early stages. Therefore, initial line dislocations arose during the formation of DNA substrates, and further impacted the subsequent line assembly.

Since introducing the composing DNA bricks of the line modules, we observed the gradual formation of DNA lines on top of the DNA substrates (Fig. 4b–d, Supplementary Fig. 15). From 2 h to 6 h, the dimensions of the assembled DNA line arrays expanded, and more DNA lines were assembled on the substrates. Notably, most of the growing DNA lines were not completed after 6 h growth, displaying partially formed lines randomly distributed on the substrate. Meanwhile, line dislocations (indicated by the white arrows in Fig. 4b–d), in particular type I, had already emerged prior to the nearby lines. For example, although only four lines (two intact lines and two partially formed) appeared on the DNA substrate at 2 h (Fig. 4b), one intact line among them contained type I line dislocation. The correct lattice extension of the neighboring DNA lines was thus blocked by the line dislocations, suggesting the competing dynamics-induced defect formation.

We did not compare the LDR values during the time-lapse analysis, considering that most of the partially formed lines assembled into intact ones after longer durations. Although we observed high-rate grain boundaries after 96-h assembly, we did not observe them during the first 6 h, which suggested that high-dimensional crystallographic defects exhibited distinct assembly orders. The 2D grain boundaries at the peripheral regions mainly formed during the latter assembly stages, after the appearance of line dislocations at the center region. This was likely because of the slower lattice extension kinetics at the peripheral regions compared to the assembly kinetics on top of the preformed substrates.

### OSE to suppress crystallographic defects

Based on the above discussions, we concluded that the *x*-direction line pitch (i.e., the sequence periodicity along *x* direction) inversely correlated with the LDR values, and was less affected by the geometries or the sequences of the lines. Therefore, to suppress the pattern defect, we scaled the line pitch while retaining a relative larger sequence periodicity using the approach of OSE.

**Fig. 5:**
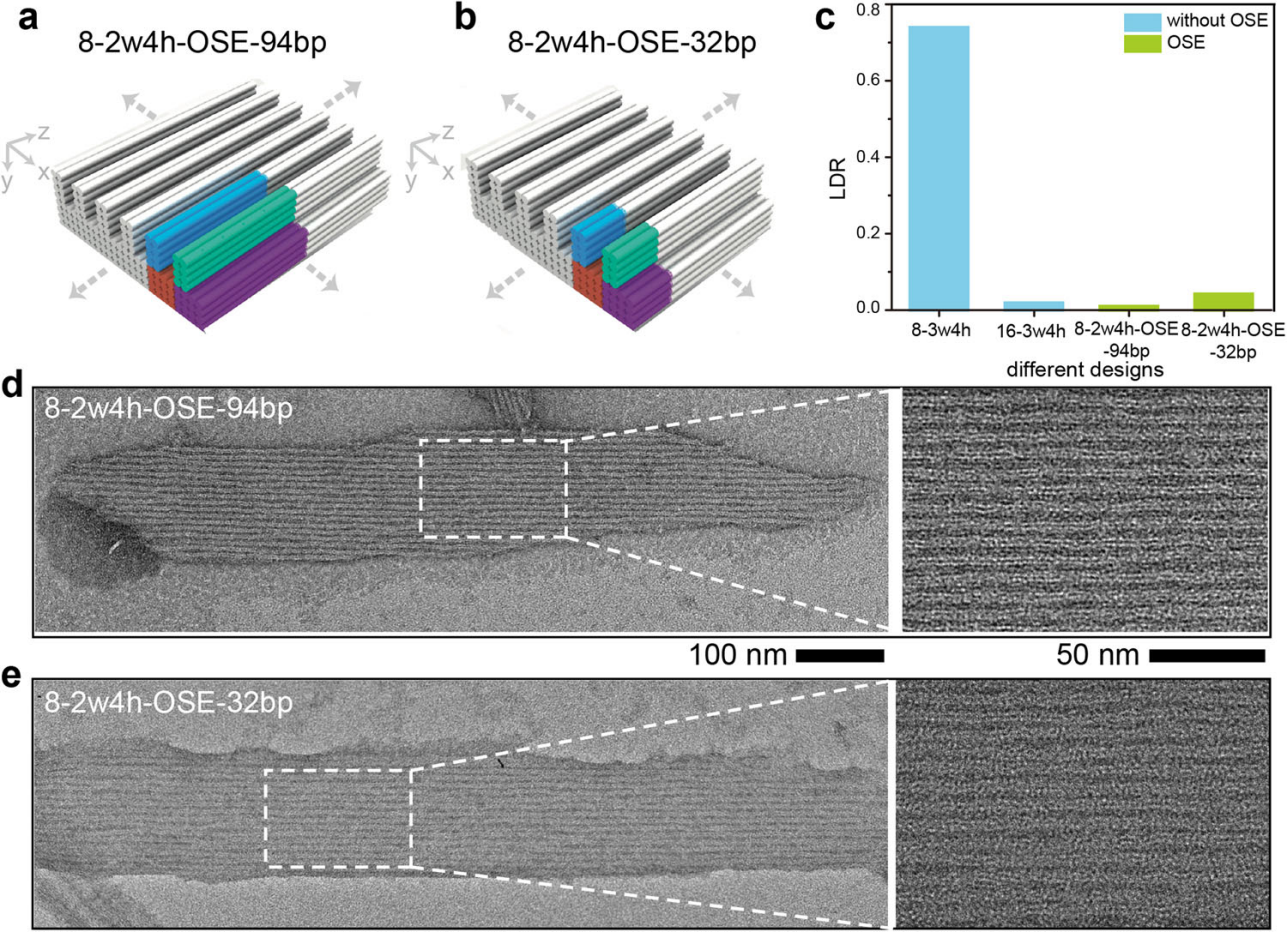
OSE approach to suppress crystallographic defects. Design schematic (**a** and **b**), zoomed-out (**d** and **e**, left) and zoomed-in (**d** and **e**, right) TEM images for the representative DNA line arrays of 8-2w4h-OSE-94bp (**a** and **d**) and 8-2w4h-OSE-32bp (**b** and **e**), where 94 bp and 32 bp denote the basepairs along *z* direction within the unit cell. The colored bundles in **a** and **b** indicate the two neighboring unit cells assigned with the orthogonal sequences. The scale bars are 100 nm (**d** and **e**, left) and 50 nm (**d** and **e**, right). **c** LDR values for different DNA line arrays designed with and without OSE approach (8-3w4h, 16-3w4h, 8-2w4h-OSE-94bp, and 8-2w4h-OSE-32bp).

To verify its effectiveness, we used DNA line arrays with designed 8.4-nm *x*-direction line pitch as an example. Specifically, we assigned orthogonal sequences (shown in Fig. 5a) for two neighboring unit cells (both using 4 helices × 4 helices × 94 basepairs for the substrate modules and 2 helices × 4 helices × 94 basepairs for the line modules, namely 8-2w4h-OSE-94bp, where 94 bp denoted the basepairs along *z* direction within the unit cell). From a viewpoint of pattern morphologies, neighboring lines were separated by 4 helices from center-to-center along *x* direction, displaying a designed line pitch of 8.4-nm (calculated from 2.1 nm diameter per dehydrated DNA helix). However, from a viewpoint of sequences periodicity, identical sequences were separated by 8 helices along *x* direction (16.8 nm designed periodicity) at a doubled line pitch. We selected 16.8 nm as the threshold of the designed sequence periodicity because of its low LDR value (as shown in Fig. 2f).

After assembly, DNA lines displayed identical 3.7 nm  ±  0.3 nm width and separated at a 7.5 nm ± 0.3 nm pitch along *x* direction (more than 100 DNA lines were counted, Fig. 5d, Supplementary Figs. 8 and 13, Table 1), similar with the design. All the DNA lines exhibited consistent assembly qualities (including width, pitch, morphology, and LDR), excluding any sequence variance-induced line defects. Although we observed slightly twisted DNA lines due to their low stiffness^42^, the overall integrity and parallel orientations of the DNA lines were not affected.

At similar line pitch, 8-2w4h-OSE-94bp exhibited a LDR value less than 1% (120 individual patterns were counted), two orders of magnitude smaller than 8-3w4h, confirming the effectiveness of OSE in suppressing the high-dimensional crystallographic defects (Fig. 5c, Supplementary Fig. 8). The minimum pitch of the defect-free DNA lines was smaller than those fabricated using current photolithography (including the extreme ultraviolet lithography)^5,63^ and directed self-assembly of block copolymers^9,64^. The remaining pattern defects arose from the grain boundaries at the peripheral regions, and typical line dislocations (as in Fig. 1b) or small DNA linkers (as in Fig. 3g) were completely absent. Notably, although designed with identical sequence periodicity, 8-2w4h-OSE-94bp exhibited smaller LDR values than that of 16-3w4h, likely because of the statistics variation of small sample size.

We further explored the effectiveness of OSE for *z*-direction sequence periodicity. We used 32 basepairs along *z* direction for both the substrate modules (i.e., 4 helices × 4 helices × 32 basepairs) and the line modules (i.e., 2 helices × 4 helices × 32 basepairs, namely 8-2w4h-OSE-32bp, Fig. 5b). Therefore, the *z*-direction periodicities were designed as 10 nm, 67% smaller than those in Fig. 5a. After assembly (Fig. 5e, Supplementary Figs. 9 and 13, Table 1), the measured line pitch was 7.6 nm ± 0.5 nm, and the line width was 3.4 nm ± 0.5 nm (more than 100 DNA line were counted). The LDR values increased by approximately five folds (i.e., 4%, 100 individual patterns were counted), compared to that of 8-2w4h-OSE-94bp (Fig. 5c). As a result, to suppress the pattern defects, OSE needed to be applied along both extension directions (i.e., *x*-*z* directions) of the periodic line arrays.

Formation of high-dimensional crystallographic defects were more dependent on the *x*-direction sequence periodicity. Along *x* direction, 11.5 nm sequence periodicity in 12-2w4h exhibited a LDR value of 22%. In contrast, in 8-2w4h-OSE-32bp, a similar 10 nm sequence periodicity value along *z* direction produced a 81% smaller LDR value (i.e., 4%). Thus, similar sequence periodicity values performed differently along *x* and *z* directions.

### Ultra-scaled metal nano-line arrays

**Fig. 6:**
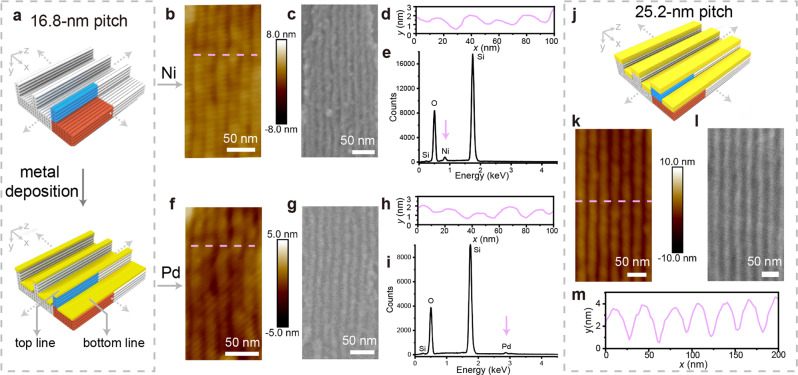
DNA-directed fabrication of ultra-scaled metal nano-line arrays. **a** Design schematic for the DNA-directed metal deposition using **16-2w4h** as template. **b**-**i** AFM images (**b**, **f**), SEM images (**c**, **g**), height profiles from AFM images (**d**, **h**), and element analysis via EDS (**e**, **i**) for the deposited Ni (**b**–**e**) and Pd (**f**–**i**) using DNA template with 16.8-nm designed line pitch, respectively. The pink dashed lines in **b** and **f** correspond to the positions for the height profiles in **d** and **h**. The scale bars in **b**, **c**, **f**, and **g** are 50 nm. Design (**j**), AFM image (**k**), SEM image (**l**), height profile from AFM image (**m**) for the deposited Pd using DNA templates with 25.2-nm designed line pitch, respectively. The pink dashed line in **k** corresponds to the position for the height profile in **m**. The scale bars in **k** and **l** are 50 nm.

Using the defect-free DNA line arrays as templates, we next fabricated parallel aligned Pd and Ni nano-lines (Fig. 6a). We first used a template with designer line pitch of 16.8 nm along *x* direction (substrate module of 8 helices × 4 helices × 94 basepairs and a line module of 2 helices × 4 helices × 94 basepairs as the repeating unit cells, namely 16-2w4h). The pre-assembled DNA line arrays were first deposited onto a Si wafer, followed by sequential rinsing with water and ethanol to remove the salt residues from surface. After that, thin-layer (2–6 nm) metal films were deposited onto the DNA patterns using thermal evaporator (for Pd) or magnetron sputtering deposition (for Ni). The deposited samples were imaged with atomic force microscope (AFM, Fig. 6b, f, Supplementary Fig. 18) and scanning electron microscope (SEM, Fig. 6c, g, Supplementary Fig. 17).

Before metal deposition, DNA lines could not be visualized under SEM due to their limited electron scattering capability. After metal deposition, we observed bright lines, corresponding to the deposited metals on top of the DNA patterns (Fig. 6c, g, more images in Supplementary Fig. 17). The chemical nature of the deposited metals was confirmed by their characteristic peaks (0.85 keV for Ni and 2.83 keV for Pd) from energy dispersive spectroscopy (EDS, Fig. 6e, i).

Under AFM measurement, both Pd and Ni displayed similar 3D morphologies on DNA templates. For instance, at metal thicknesses of 2–3 nm, the statistical analysis (more than 100 lines were counted, Supplementary Fig. 19) revealed similar critical dimensions before and after metal depositions. The measured widths along *x* direction of the top metal lines were around 8–13 nm, and the depths (thickness differences between high and low positions) were around 1–3 nm along *y* direction (Fig. 6d, h, more images in Supplementary Fig. 18). Notably, the width of the top metal line was similar to that of bare DNA lines without metal deposition (Supplementary Fig. 19), and larger than that observed in SEM (Fig. 6c, g). This observation was due to AFM tips blocked by the sidewall of templating DNA lines, and consistent with previous report^38^. Nevertheless, the 3D feature of DNA templates was well retained in the deposited metal patterns, which revealed the nondestructive nature of the reproducible metal deposition to DNA templates.

By programming the line pitch of DNA templates, we further demonstrated Pd nano-line arrays using DNA templates with prescribed ~25.2-nm line pitch (Fig. 6j–m). Metal nano-line arrays displayed width ~20-nm line width and 1–3 nm line depths. Cross-section analysis indicated that the lateral metal growth was confined by DNA sidewalls, and not penetrating into DNA lattices. The thicknesses of the metal layers impacted their resistance against the external stresses, which further determined the deformation of 3D cross-section morphologies by focused ion beam milling (Supplementary Figs. 20 and 21).

Different metals exhibited distinct deposition qualities on the top of the DNA templates. For metals such as gold, aluminum, and silver, we only observed cracked films and randomly formed nanoparticles on the DNA templates, instead of linear morphologies (Supplementary Fig. 16). Different surface adhesion of metal precursors on DNA lattices may lead to the distinct deposition qualities. For example, less adhered metal atoms tended to form discrete nanoparticles instead of extending nano-lines; meanwhile the uneven metal nanoparticle growth could promote surface tension and lead to the film cracks.

## Discussion

Periodic DNA sequences construct micron-scale 3D DNA templates from simplified components. However, periodic sequences may also induce lattice cross-talk between neighboring unit cells, which is inversely correlated with their periodicity. Such inverse correlation arises from the dynamic competing between the penetration of defective lattices and the assembly of correct lattices pathways. Diffusion kinetics of individual DNA bricks accelerates from 15.3-nm sequence periodicity to 8.2-nm sequence periodicity, owing to the higher concentration of DNA bricks at 8.2-nm periodicity. However, the diffusion kinetics contributes equally to both the penetration of defective lattices and the assembly of correct lattices, and does not selectively activate defect penetration. Meanwhile, at smaller periodicities, because it takes less time for the defective lattices penetrating into neighboring lattices, the disproportional increase of the corresponding LDR value emerges; whereas at larger periodicities, the lattice penetration dynamics is largely slowed down, and the dominating correct lattice assembly lowers the corresponding LDR values. Thus, the rapid changes of the defect penetration dynamics towards sequence periodicity alter the competing consequence of multiple dynamic pathways. Different line designs have little impacts to the LDR, as results of their similar dynamics regarding the lattice assembly and the defect penetration pathways.

Scaling the line pitches while retaining the minimal sequence periodicity is thus necessary for defect-free ultra-scaled DNA arrays. Based on this consideration, we suggest the OSE approach, which introduces heterogeneous sequences into neighboring unit cells, to demonstrate ultra-scaled 7.5-nm line pitch with LDR less than 1%. Because identical sequenced DNA bricks are separated by a periodicity of 15 nm, their cross-talk is effectively suppressed by almost two orders of magnitude. Meanwhile, heterogeneous sequences do not impact the overall integrity of neighboring lines. Current DNA templates still exhibited wide dimension distributions along *x*-*z* directions and irregular leaf-like morphologies, owing to the absence of edge confinement during lattice extensions. However, such unevenness could be addressed in principle via the introduction of edge protectors^43,59,65^ or fully-addressable DNA building blocks^33^, as well as hieratically assembled DNA origamis^66–69^, where specific dimensions could be precisely designed.

Except the requirement of the minimal sequence periodicity, OSE does not introduce other constrains to the designs of DNA building blocks nor their sequences. Thus, OSE is compatible with diverse DNA self-assembly strategies and design softwares, besides DNA brick crystals adopted here. Furthermore, without the need of optimizing the assembly or the separation conditions in different 3D patterns, OSE provides a generalizable method for suppressing the high-dimensional defects; whereas, conventional defect eliminations via thermal annealing^58^ or the rate-zonal centrifugation^61^ typically require complex optimizations tailored to each specific pattern.

Besides the self-assembly of micron-scale DNA lattices, OSE may also be applied in the directed assembly of block copolymers or protein lattices^70^. By assigning different chemical interactions into the neighboring proteins or block copolymers, selectively reduced lattice penetration dynamics may suppress their high-dimensional crystallographic defects. Notably, although we selected 15 nm as the threshold of the sequence periodicity, other self-assembly systems may adopt distinct threshold values according to their chemical nature and kinetic differences.

Scalable construction of ultra-scaled defect-free DNA templates enables the programmable nano-fabrication of functional materials down to sub-10 nm critical pitches, promoting size-dependent quantum confinement. It may be further possible to be scaled to 6 nm pitch, where coherent quantum transports arise. Therefore, besides the widely-explored semiconductors and metals, quantum materials templated on the defect-free DNA templates could enable complex quantum transport characteristics, including tunneling transistors and spintronics. Furthermore, toward the large-area uniformity requirement in the scalable nano-fabrications, hierarchical surface assembly will align micron-sized DNA templates with uniform size into the periodic arrays^43–45^, and break the size limitation of individual DNA templates.

## Methods

### Sample preparation

All DNA bricks were purchased from Sangong Biotech (Shanghai, China). To assemble DNA structures, unpurified DNA bricks were first mixed to a final concentration ranging from 200 nM to 800 nM (depending on the total numbers of DNA bricks for each design) in buffer (1 ×  TE buffer, containing 5 mM Tris and 1 mM EDTA at pH 7.9, 40 mM MgCl_2_).

### Annealing protocol

We used DNA modular epitaxy to control the assembly pathway. In brief, the DNA substrate module mixtures were sequentially cooled from 85 °C for 15 min, 44 °C for 12 h, 39 °C for 24 h, and finally at 31 °C for 8 h. Next, the DNA mixtures (DNA line modules, substrate modules, mixed with the pre-assembled substrate modules) were incubated sequentially at 39 °C for 48 h and 33 °C for 48 h. The samples were cooled at 4 °C for further characterization or assembly without any further purification.

### TEM imaging

The carbon-coated TEM grids were first glow-discharged for 30 s at 5 mA electric current using PELCO easiGlow Discharge system (Ted Pella Inc., USA). The 4 µL DNA line array was adsorbed for 4 min onto the TEM grids, then was dried by Whatman filter paper. Next, the DNA line array was sequentially stained for 7 s using a 2% (wt/vol) aqueous uranyl formate solution, and then was dried by Whatman filter paper. Finally, TEM imaging was performed using a JEOL JEM-2100 TEM operated at 120 kV.

### DNA deposition on Si substrate

The 0.5 µL 16.8-nm or 25.2-nm pitch DNA line array was first diluted using 5.5 µL 15 mM MgCl_2_. At the same time, the silicon substrate was glow discharged for 30 s at 50 W power (Minilock-phantom III, Trion). Next, the 6 µL diluted sample was deposited onto Si substrate, then 0.5 µL NiCl_2_ (10 mM) subsequently added in the solution. After 60 min deposition, the solution was blown away by the nitrogen gas. Finally, the substrate was sequentially rinsed in 75%, 90%, and 100% (vol/vol) ethanol (5 s per step).

### Metal deposition on DNA line array

2–6 nm thickness of palladium (Pd) was deposited on the DNA line array by the thermal evaporation method at 0.3 Å/s rate (DE400, DeTech). 4-nm thickness of nickel (Ni) was deposited by the magnetron sputtering deposition system for 20 s at 100 W power (PVD 75, Kurt J Lesker Inc, USA).

### AFM imaging

The DNA templated metal nano-line array prepared above was imaged by AFM (Bruker, Multimode 8) via tapping mode with tip (Bruker OTESPA-R3).

### SEM imaging

The DNA templated metal nano-line array prepared above was imaged by SEM (S-4800, Hitachi) at 5 V, 10 mA. The EDS signal was collected by SEM at 10 V, 20 mA.

## Supplementary information

Supplementary Information

## Data Availability

All data generated in this study are included in this published article and in Supplementary Information files. All the relevant data for this study can also be accessed from the corresponding authors upon request.
