# Supplementary material for: Suppressing high-dimensional crystallographic defects for ultra-scaled DNA arrays
Source: Nat Commun. 2022 May 16;13:2707. doi: 10.1038/s41467-022-30441-1 (PMC9110747; doi:10.1038/s41467-022-30441-1)
Supplement: Supplementary file 1 — Supplementary Information [file 41467_2022_30441_MOESM1_ESM.pdf]

Supplementary Information

Suppressing high-dimensional crystallographic defects for ultra-scaled DNA arrays

Yahong Chen[1,2], Chaoyong Yang[1,3], Zhi Zhu[*1], Wei Sun[*2]

1 Collaborative Innovation Center of Chemistry for Energy Materials, The MOE Key Laboratory of Spectrochemical Analysis and Instrumentation, State Key Laboratory of Physical Chemistry of Solid Surfaces, Key Laboratory for Chemical Biology of Fujian Province, Department of Chemical Biology, College of Chemistry and Chemical Engineering, Xiamen University, Xiamen, 361005, China

2 Key Laboratory for the Physics and Chemistry of Nanodevices and Center for Carbon-Based Electronics, School of Electronics, Peking University, Beijing 100871, China

3 Institute of Molecular Medicine, Renji Hospital, School of Medicine, Shanghai Jiao Tong University, Shanghai, 200127, China

E-mail address*: sunw@pku.edu.cn, zhuzhi@xmu.edu.cn

Contents

S1 TEM images of different designs. 3

S1.1 TEM images of 8-3w4h 3
S1.2 TEM images of 12-3w4h 3
S1.3 TEM images of 16-3w4h 4
S1.4 TEM images of 12-2w4h 4
S1.5 TEM images of 12-4w4h 4
S1.6 Design and TEM images of 8-4w4h 5
S1.7 TEM images of 8-2w4h-OSE-94bp 6
S1.8 TEM images of 8-2w4h-OSE-32bp 7

S2 Summary about the defect compositions of the designed structures 8

S3 The DNA line pitch and width distributions of different designs 9

S3.1 DNA line pitch distributions 9
S3.2 Measuring the DNA line pitches and widths for different designs 10

S4 Structure evolving at different growth durations 11

S4.1 Growth time at 0 h 11
S4.2 Growth after 2 h, 4 h and 6 h 11

S5 Metal nano-line arrays 12

S5.1 SEM image after different metal deposition on DNA pattern 12
S5.2 AFM image after different metal deposition on DNA pattern 13
S5.3 Statistical evaluation about the reproducibility of the metal pattern 14
S5.4 The method for cross-sectional TEM characterization. 15
S5.5 Cross-section TEM for metal nano-line arrays 16

S1 TEM images of different designs.

S1.1 TEM images of 8-3w4h

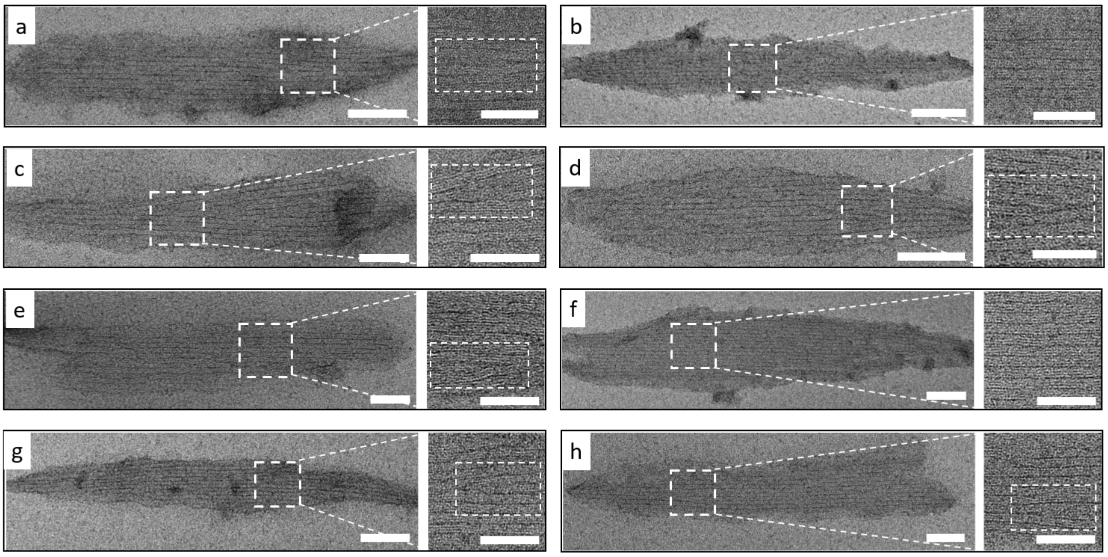

Supplementary Figure 1. TEM images of 8-3w4h structures. (a-h) Left panels (zoomed-out) showed the morphologies of DNA line arrays with 8.2-nm line pitch, scale bar: 100 nm. Right panels showed the zoomed-in morphologies, scale bar: 50 nm. Among them, only (b) and (f) were correctly assembled DNA line arrays, and the others were defective DNA line arrays. The dash white boxes in the zoom-in morphologies indicated the defective areas.

S1.2 TEM images of 12-3w4h

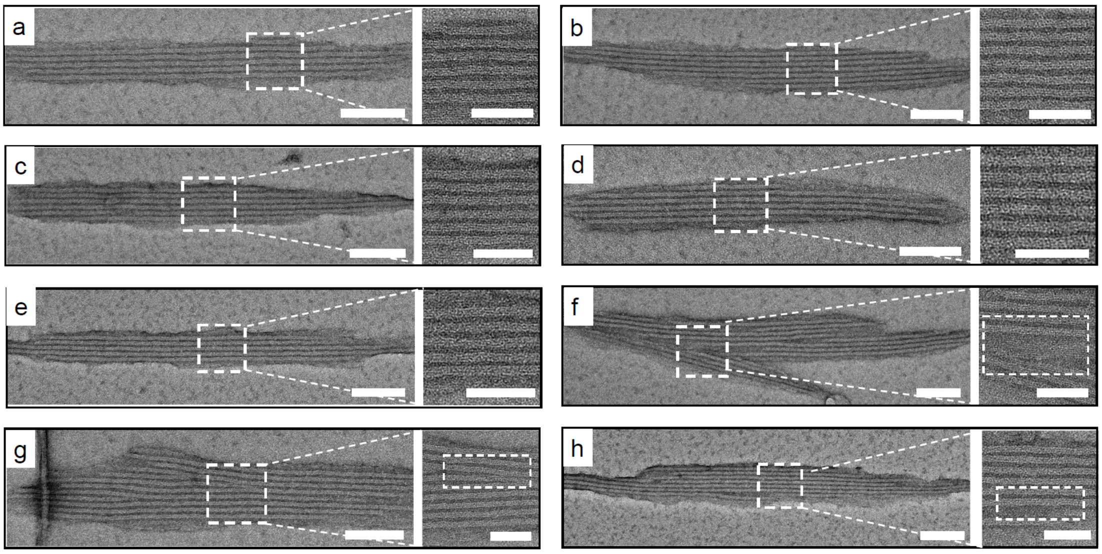

Supplementary Figure 2. TEM images of 12-3w4h structures. (a-h) Left panels (zoomed-out) showed the morphologies of DNA line arrays with 11.5-nm line pitch, scale bar: 100 nm. Right panels showed the zoomed-in morphologies, scale bar: 50 nm. Among them, (a-e) were correctly assembled DNA line arrays, (f-h) were defective DNA line arrays. The dash white boxes in the zoomed-in images indicated the defective areas.

S1.3 TEM images of 16-3w4h

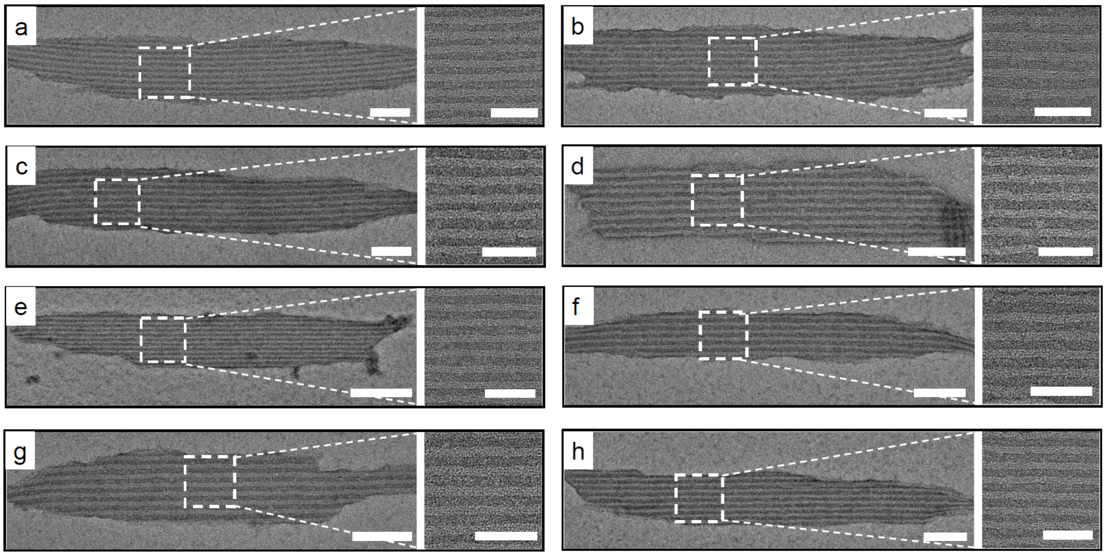

Supplementary Figure 3. TEM images of 16-3w4h structures. (a-h) Left panels (zoomed-out) showed the correct morphologies of DNA line arrays with 15.3-nm line pitch, scale bar: 100 nm. Right panels showed the zoomed-in morphologies, scale bar: 50 nm.

S1.4 TEM images of 12-2w4h

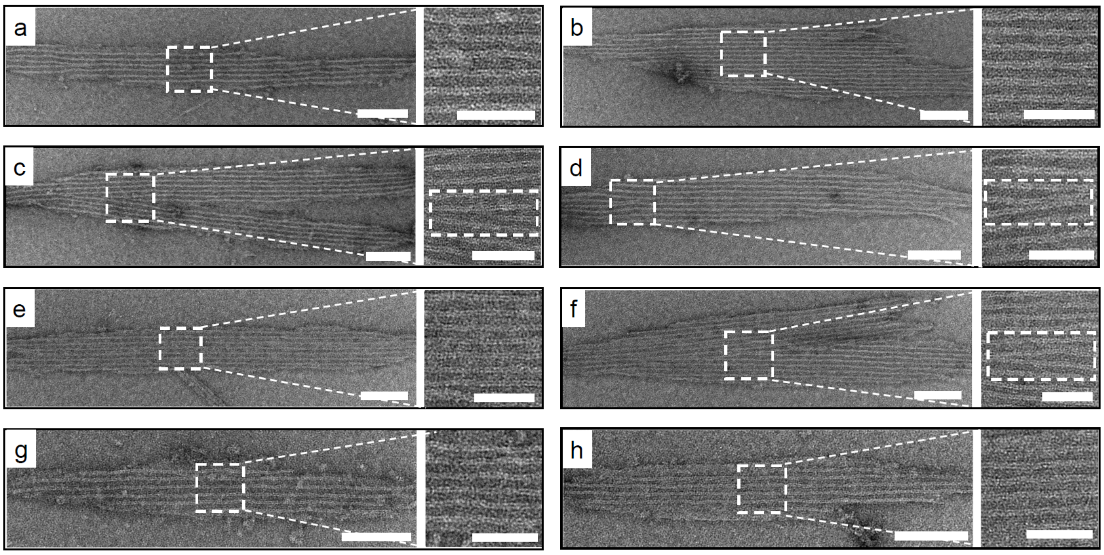

Supplementary Figure 4. TEM images of 12-2w4h structures. (a-h) Left panels (zoomed-out) showed morphologies of DNA line arrays with 11.6-nm line pitch, scale bar: 100 nm. Right panels showed the zoomed-in morphologies, scale bar: 50 nm. Among them, (a-b, e, g-h) were correctly assembled DNA line arrays, (c, d, f) were defective line arrays. The dash white boxes in the zoomed-in images indicated the defective areas.

S1.5 TEM images of 12-4w4h

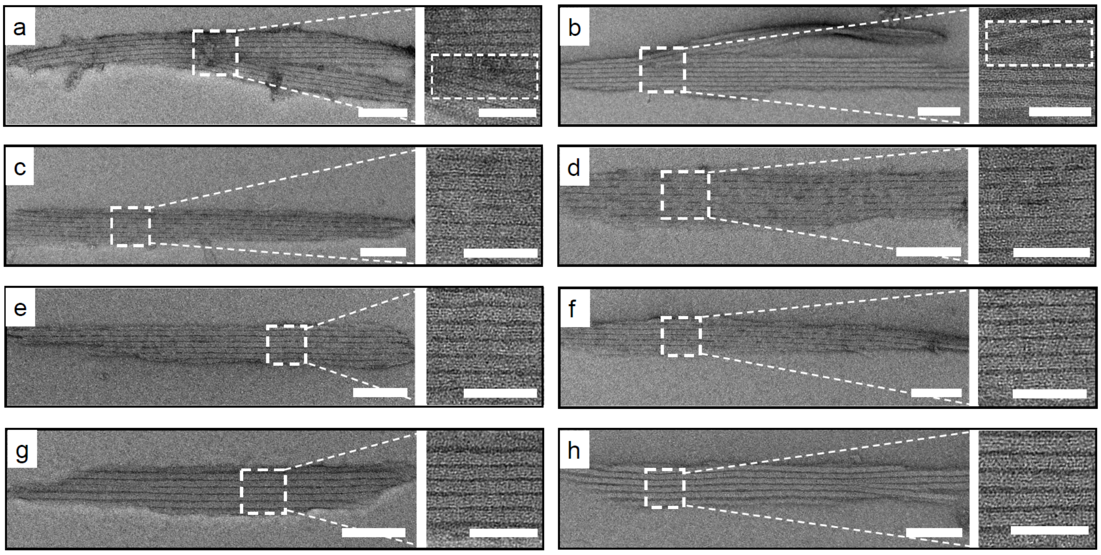

Supplementary Figure 5. TEM images of 12-4w4h structures. (a-h) Left panels (zoomed-out) showed the morphologies of DNA line arrays with 11.3-nm line pitch, scale bar: 100 nm. Right panels showed the zoomed-in morphologies, scale bar: 50 nm. Among them, (a-b) were defective DNA line arrays, (c-h) were correctly assembled DNA line arrays. The dash white boxes in the zoomed-in images indicated the defective areas.

S1.6 Design and TEM images of 8-4w4h

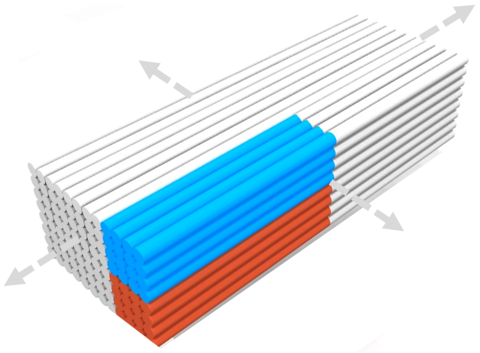

Supplementary Figure 6. Schematic of the 8-4w4h design. The orange color showed the substrate module, and the blue color showed the DNA line module.

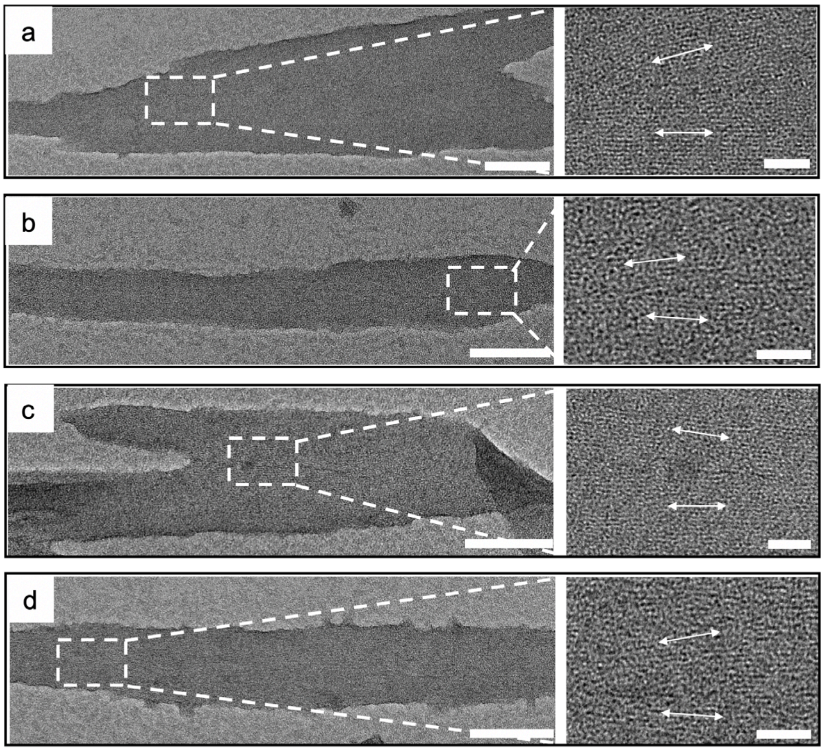

Supplementary Figure 7. TEM images of 8-4w4h structures. (a-d) Left panels (zoomed-out) showed the defective morphologies of DNA line arrays with 8-nm pitch, scale bar: 100 nm. Right panels showed the zoomed-in morphologies, scale bar: 20 nm. The white arrows in the zoomed-in morphologies indicated the orientation of the DNA helix.

S1.7 TEM images of 8-2w4h-OSE-94bp

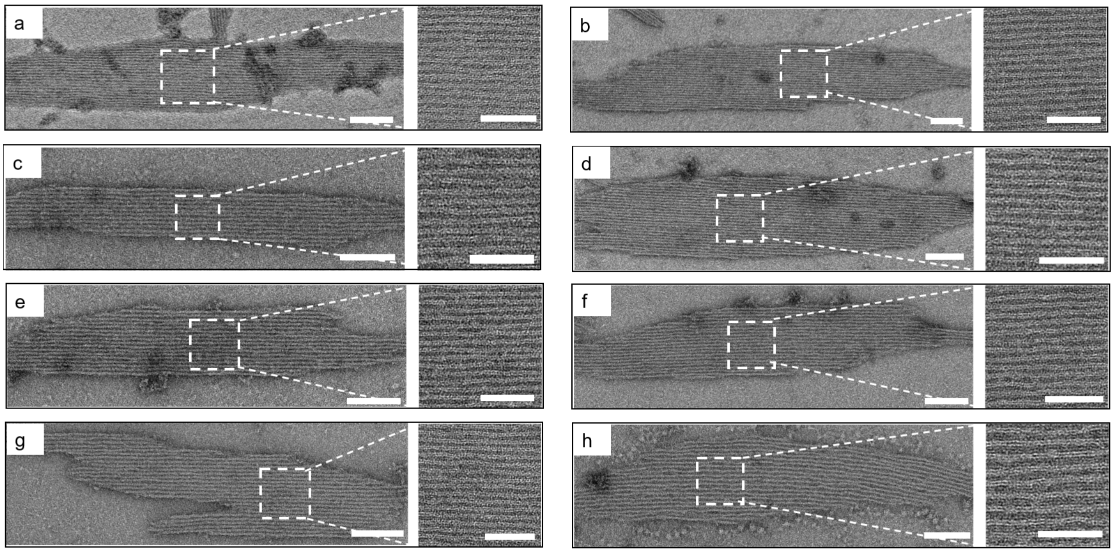

Supplementary Figure 8. TEM images of 8-2w4h-OSE-94bp structures. (a-h) Left panels (zoomed-out) showed the morphologies of DNA line arrays with 7.5-nm line pitch, scale bar: 100 nm. Right panels showed the zoomed-in morphologies, scale bar: 50 nm.

S1.8 TEM images of 8-2w4h-OSE-32bp

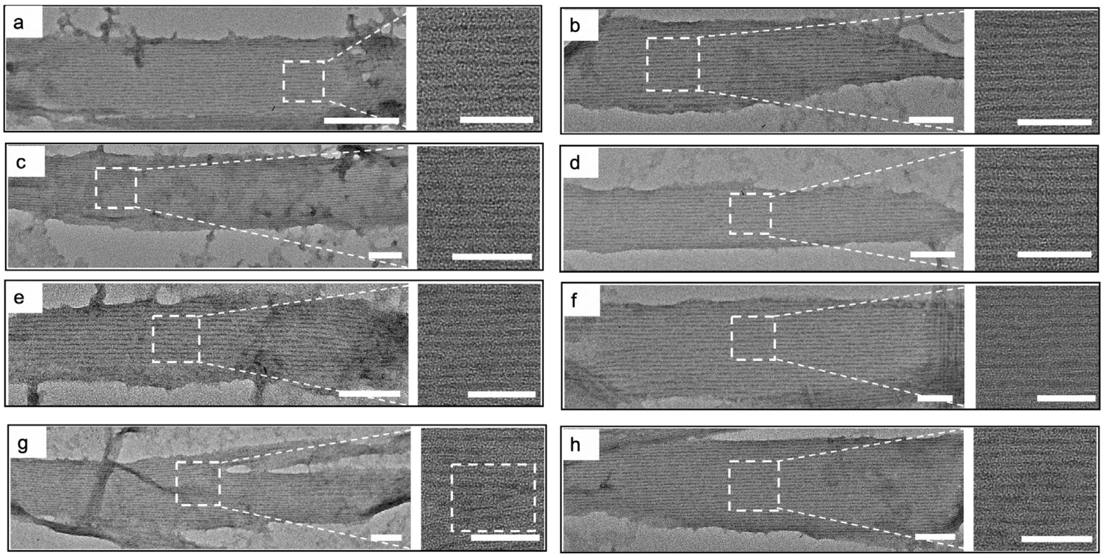

Supplementary Figure 9. TEM images of 8-2w4h-OSE-32bp structures. (a-h) Left panels (zoomed-out) showed the morphologies of DNA line arrays with 7.5-nm line pitch, scale bar: 100 nm. Right panels showed the zoomed-in morphologies, scale bar: 50 nm. Among then, (a-f, h) were correctly assembled DNA line arrays. (g) was defective DNA line array. The dash white boxes in the zoomed-in images indicated the defective areas.

S2 Summary about the defect compositions of the designed structures

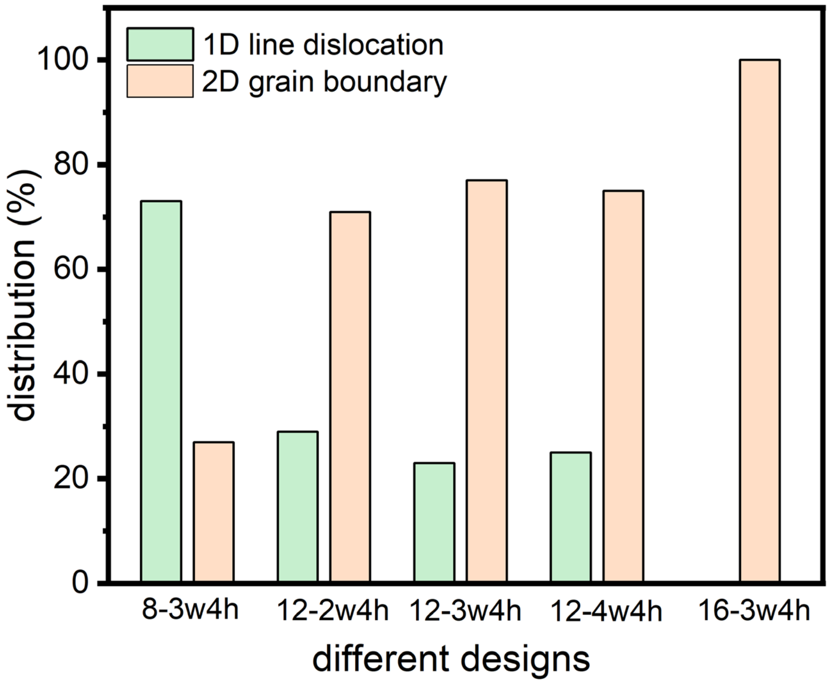

Supplementary Figure 10. Histogram about the distributions of 1D line dislocation (green) and 2D grain boundary (yellow) for different designs (from left to right: 8-3w4h, 12-2w4h, 12-3w4h, 12-4w4h and 16-3w4h designs)

S3 The DNA line pitch and width distributions of different designs

S3.1 DNA line pitch distributions

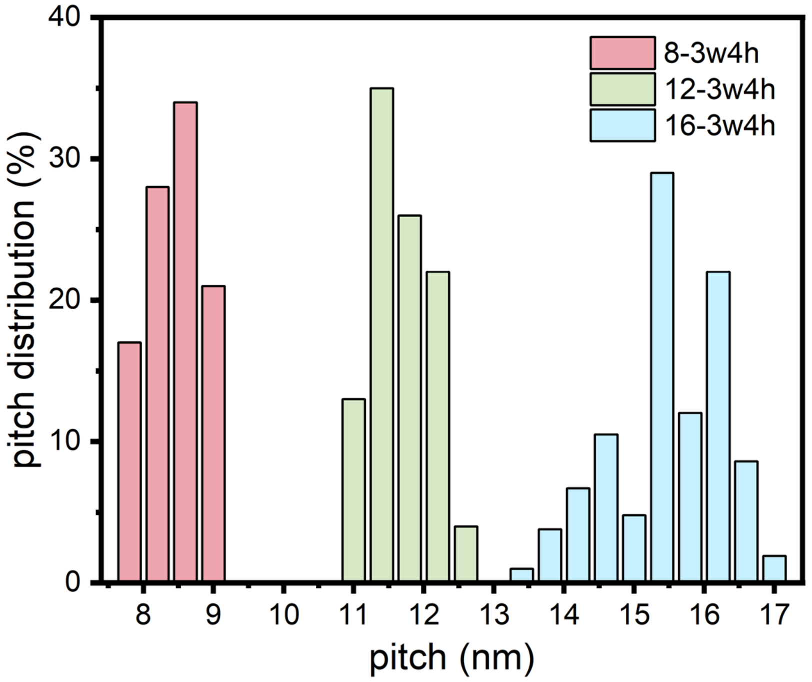

Supplementary Figure 11. The line pitch distributions for the 8-3w4h (pink), 12-3w4h (green), and 16-3w4h (blue) designs.

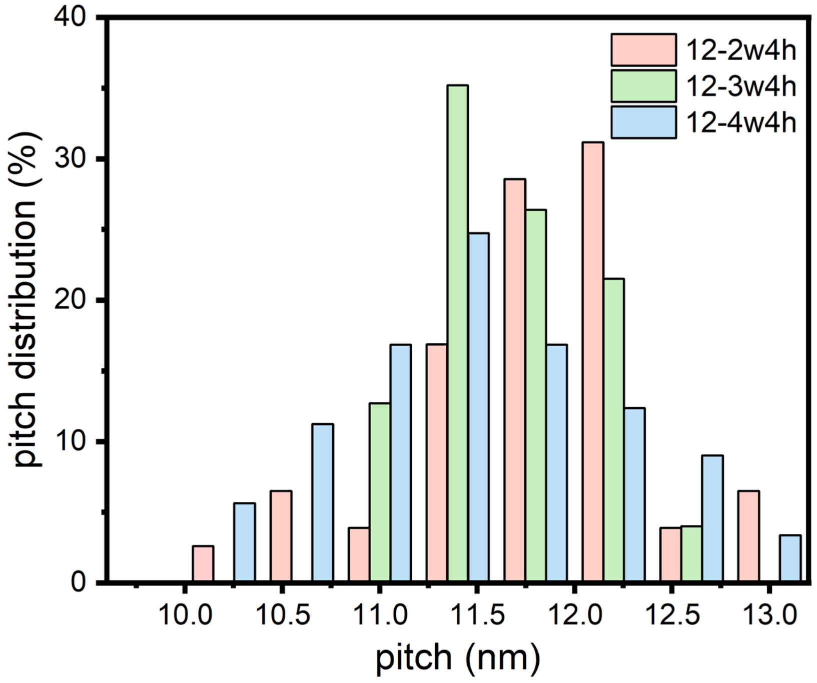

Supplementary Figure 12. The line pitch distributions of the 12-2w4h (pink), 12-3w4h (green), and 12-4w4h (blue) designs.

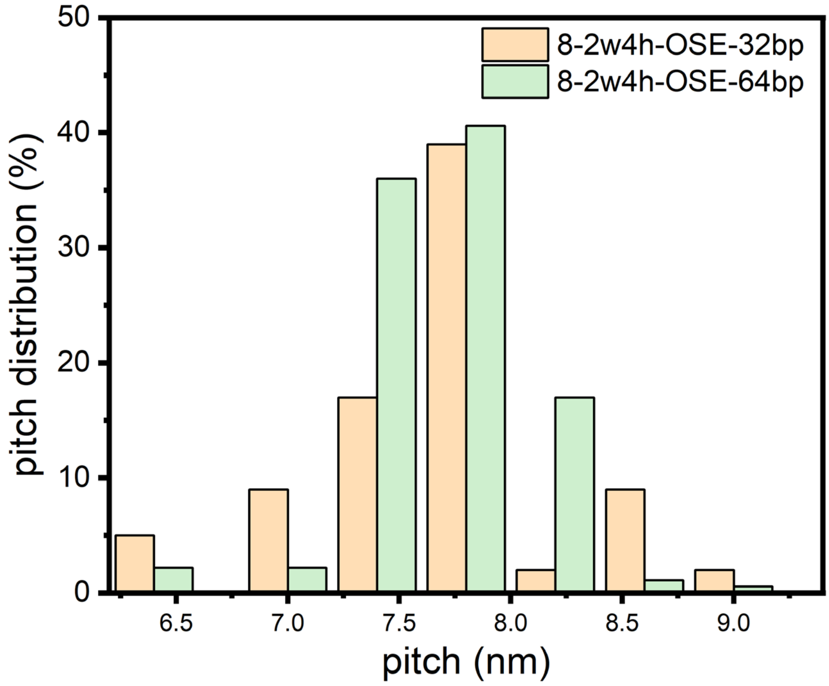

Supplementary Figure 13. The line pitch distributions of the 8-2w4h-OSE-32bp (orange) and the 8-2w4h-OSE-94bp (green) designs.

S3.2 Measuring the DNA line pitches and widths for different designs

Design	Line Pitch/nm	Line width/nm
8-3w4h	8.16±0.42	6.38±0.47
12-2w4h	11.61±0.61	3.93±0.28
12-3w4h	11.47±0.44	6.63±0.19
12-4w4h	11.30±0.69	7.90±0.33
16-3w4h	15.26±0.60	6.86±0.27
8-2w4h-OSE-94bp	7.52±0.32	3.74±0.31
8-2w4h-OSE-32bp	7.58±0.52	3.42±0.52

Supplementary Table 1. The average numbers of different DNA line pitches and widths counted from 100–200 DNA lines for each design.

S4 Structure evolving at different growth durations

S4.1 Growth time at 0 h

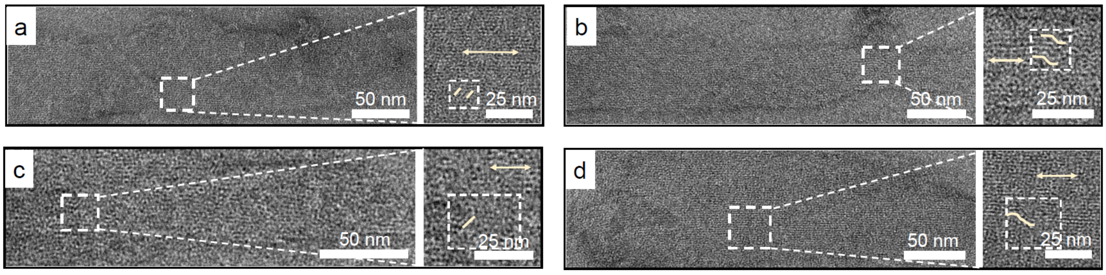

Supplementary Figure 14. TEM images of the DNA substrate for 8-3w4h structure. (a-d) Left panels (zoomed-out) showed the morphologies of DNA substrate growth at 0 h. Right panels showed the zoomed-in morphologies. In the zoomed-in morphologies, the white dash box indicated defect regions, the yellow arrows indicated the correct orientation of DNA helices, and the yellow curve line indicated the ssDNA strand dislocated at the neighboring area.

S4.2 Growth after 2 h, 4 h and 6 h

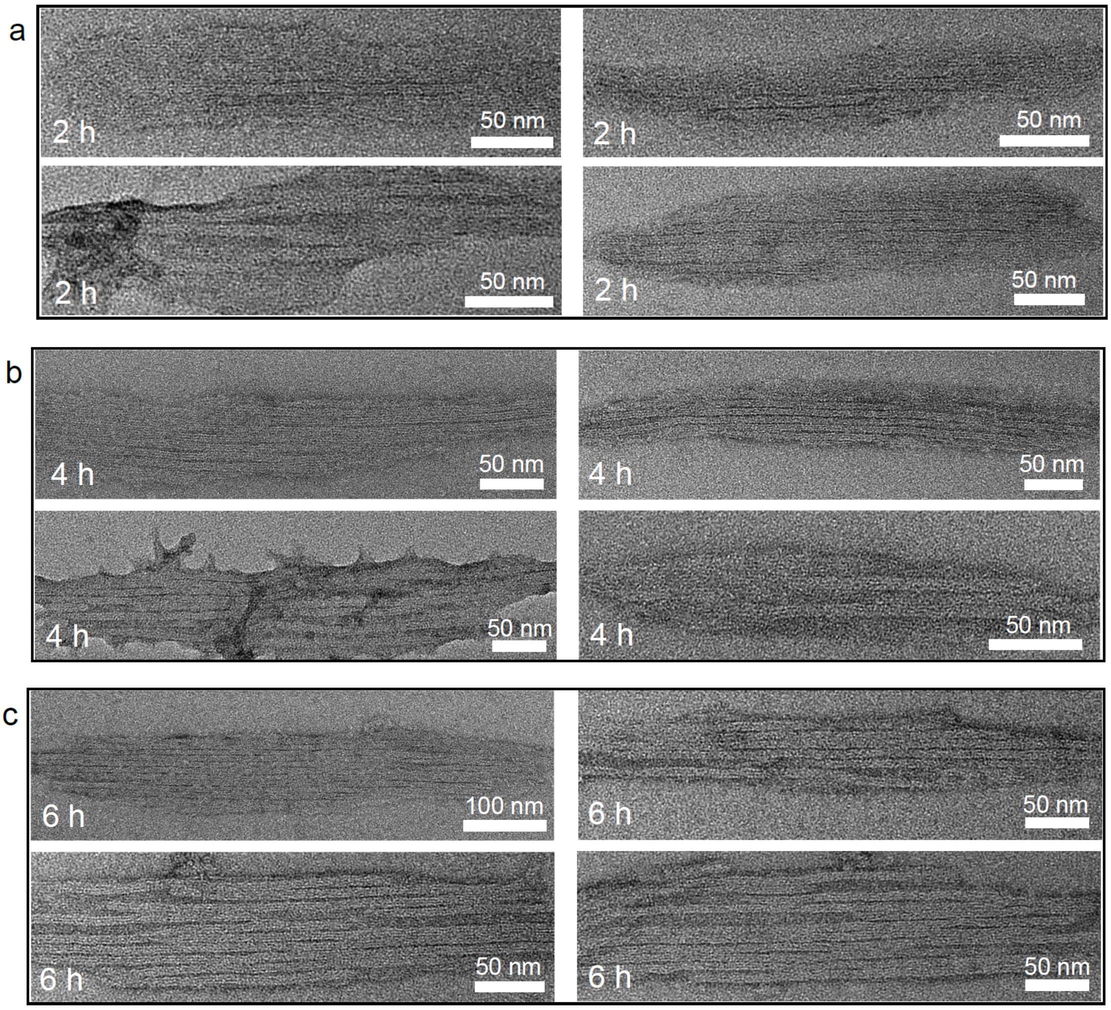

Supplementary Figure 15. TEM images about DNA line arrays assembly at 2 h (a), at 4h (b), 6h (c).

S5 Metal nano-line arrays

S5.1 SEM image after different metal deposition on DNA pattern

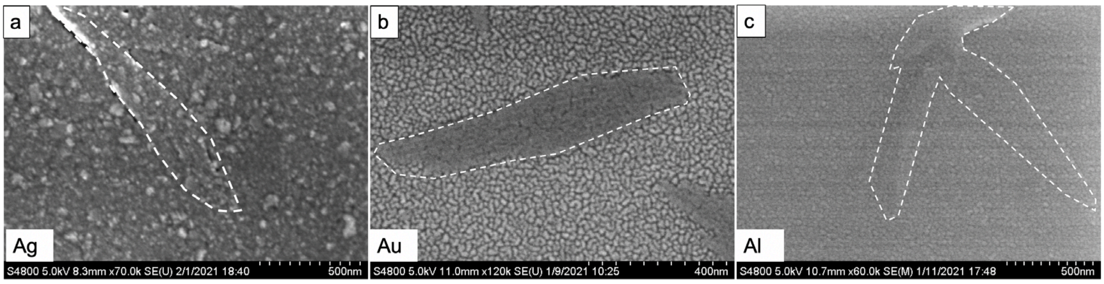

Supplementary Figure 16. SEM images of 4-nm thick Ag (a), Au (b) and Al (c) deposited onto DNA templates with 16.8-nm line pitches.

The 4-nm thick Ag film was deposited by magnetron sputtering, and the 4-nm thick Au and Al film were deposited by thermal evaporation. The SEM images displayed cracked films and randomly formed nanoparticles on the surface of DNA templates, indicating that these metals could not conformally adhere to the DNA surface.

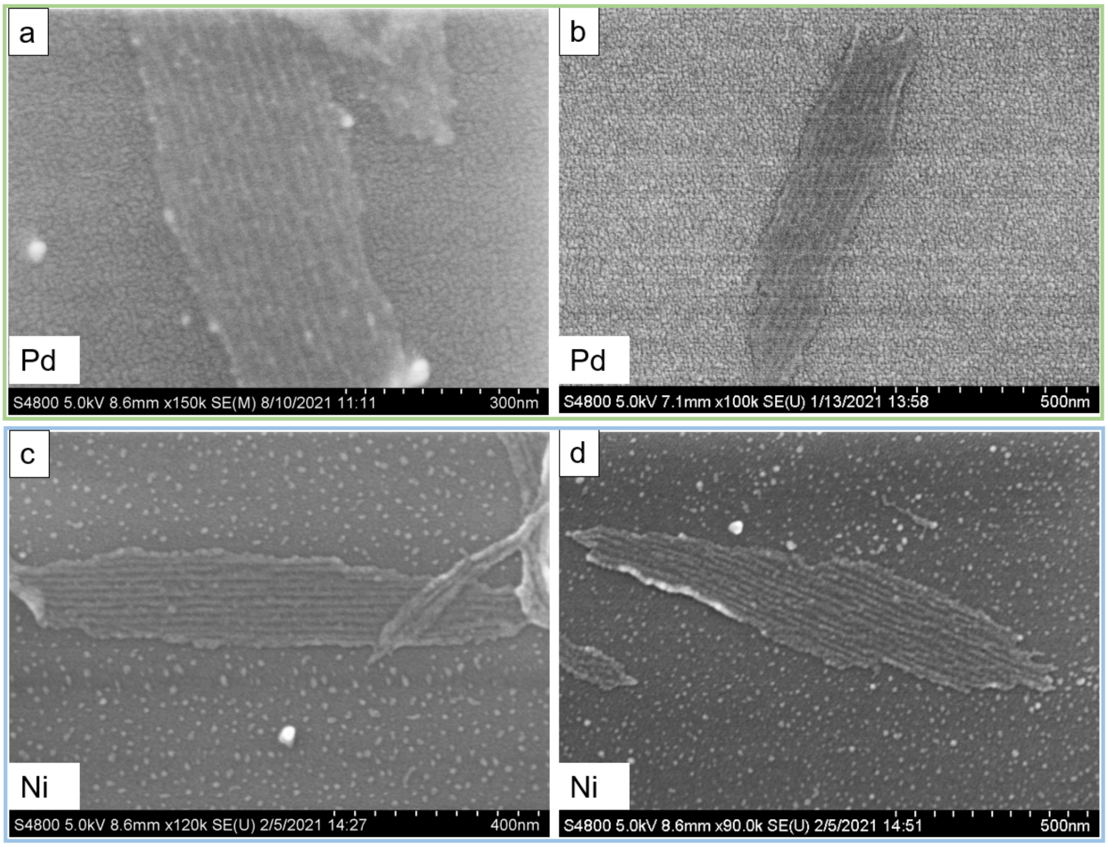

Supplementary Figure 17. SEM images of the 4-nm thick Pd (a-b) and Ni (c-d) deposited on the DNA templates with 16.8-nm line pitch.

S5.2 AFM image after different metal deposition on DNA pattern

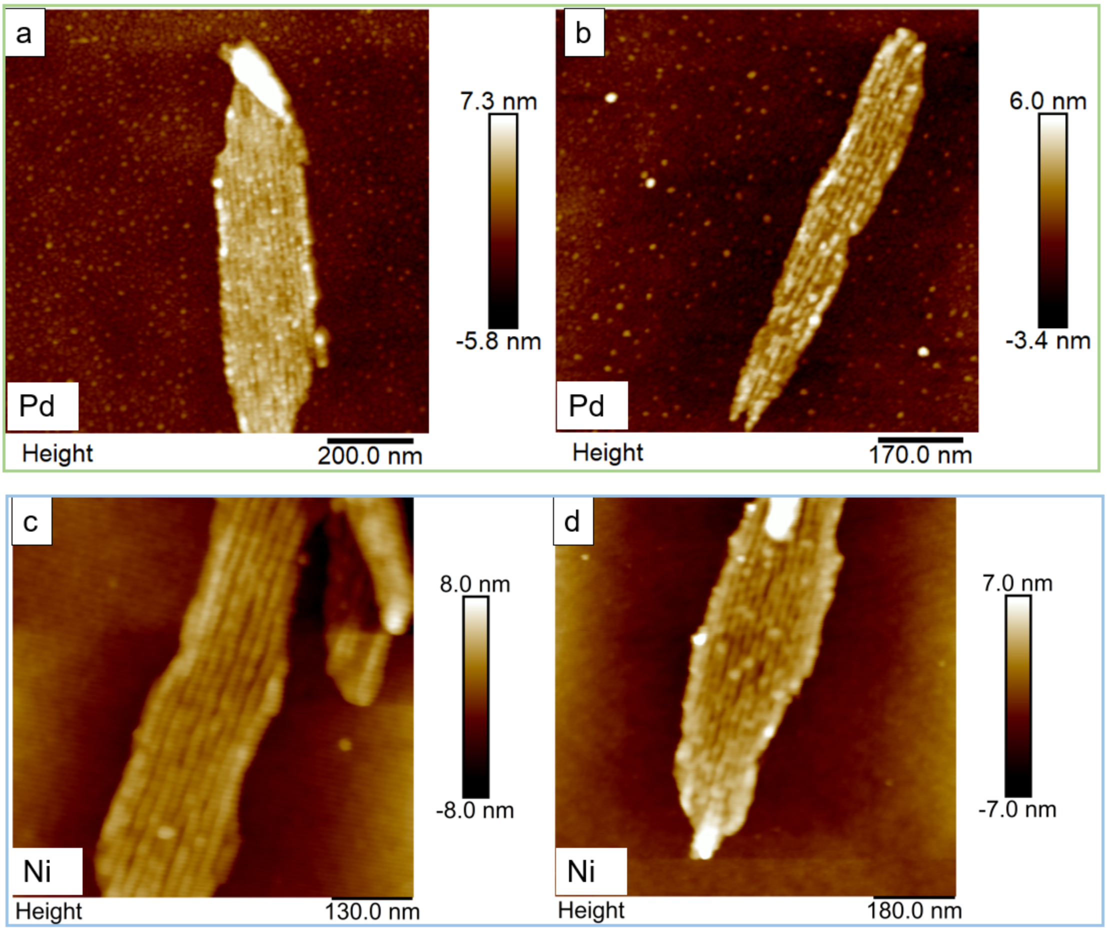

Supplementary Figure 18. AFM images of 4-nm thick Pd (a-b) and Ni (c-d) deposited onto the DNA templates with 16.8-nm line pitches.

S5.3 Statistical evaluation about the reproducibility of the metal pattern

In order to statistically evaluate the reproducibility of the metal patterns, 2~3 nm thicknesses of Pd were deposited on the DNA template with 25.2-nm pitch or 16.8-nm pitch. We separately counted up to 100 lines before and after Pd deposition under AFM measurement. The statistical analysis indicated that (Supplementary Fig.19), before and after metal depositions, the critical pattern dimensions (including the line width, line pitch, and the spacing) remained similar values.

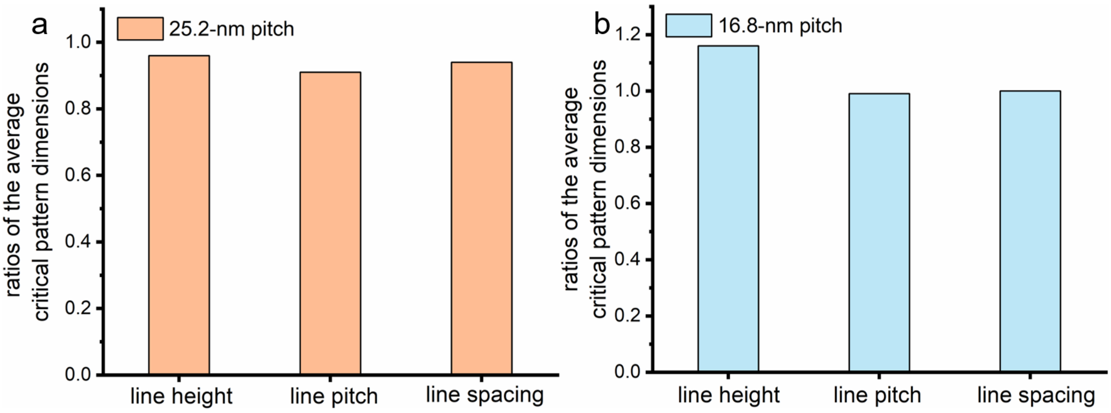

Supplementary Figure 19. The ratio of the average critical dimensions before and after metal deposition for (a) 25.2-nm DNA pitch and (b) 16.8-nm DNA pitch. The values were obtained via AFM measurements. Up to 100 lines before and after Pd deposition were counted.

S5.4 The method for cross-sectional TEM characterization.

To understand the morphology of mental layer coating within the nanotrench, we prepared samples using FEI Helios G4 UX DualBeam focused ion beam (FIB)/SEM system. To protect the interested samples, 200–300 nm depth of carbon and 2 μm depth of platinum were deposited sequentially under electron and ion beams. Then the sample was milled about 12 μm depth using the Ga ion beam, forming the U-cut. Lamella was extracted from the substrate into copper TEM grid through lift-out method. Finally, the lamella was thinned down by ion beam at 30 kV and polished at 3 kV. The cross-sectional images were imaged by TEM (FEI Tecnai F20) at 200 kV

S5.5 Cross-section TEM for metal nano-line arrays

We used the DNA templates with 25.2-pitch as a model system for the FIB milling and the cross-sectional analysis. We first coated the surface-deposited DNA templates with four different thicknesses of Pd layer (6 nm, 3.7 nm, 2.5 nm, and 2 nm), and evaluated the groove depths using AFM (up to 100 lines were counted). For subsequent FIB milling, we sequentially deposited 200 nm amorphous carbon and more than 2 microns Pd layer onto the metal-coated DNA templates to protect the beneath structures from the high-energy ion milling process, followed by cutting the sample along the *x-y* directions. Then we imaged the *x-y* cross-section morphologies via TEM (Supplementary Fig. 21a). Notably, to enhance the structural rigidity of DNA templates, we used Ni^{2+} to treat the DNA templates prior to their surface deposition

We observed that, before FIB milling, the DNA-templated Pd patterns exhibited similar groove depths (~2 nm) to that of pure DNA templates (Supplementary Fig. 20), regardless of the metal thicknesses. Thin-layer Pd coating did not affect either the pattern integrity or the groove depths of DNA templates, which was in consistent with the previous reply to the reviewer.

However, after FIB milling, we observed a metal thickness-dependent variation of the cross-section morphologies (Supplementary Fig. 21). When the metal thicknesses (6 nm and 3.7 nm) exceeded the typical depths of DNA grooves (~2 nm), metals layers on top of the DNA lines and within the DNA grooves connected from the side, producing the continuous ripple-like periodic films (Supplementary Fig. 21 a, b and c). Notably, the lateral metal growth within the DNA grooves were confined by the sidewalls of DNA lines, displaying similar dimensions and periodicities. The sidewalls DNA lines also prevented the lateral metal growth from penetrating into the lattices of DNA lines. The faint shades around the metal layer were likely to be the residuals at the cut edge during FIB milling.

Decreasing the metal thicknesses from 6 nm to 3.7 nm led to smaller groove depths from 3 nm (at 6-nm metal thickness) to ~1.7 nm (at 3.7-nm metal thickness) (Supplementary Fig. 20), indicating more stresses pressed onto the metal ripples. At thinner metal thicknesses (2.5 nm and 2 nm), the periodic 3D morphologies vanished (Supplementary Fig. 21 a, d and e). The groove depths decreased from less than 1 nm at 2.5-nm metal thickness to zero nm at 2-nm metal thickness (which is the minimal value we could deposit with our deposition facilities), leaving only planar morphology.

Considering that such planar morphologies were not observed in the AFM images before FIB milling, we therefore ascribed these morphological changes mainly to the template destruction during FIB milling, rather than during DNA-templated metal patterning. FIB milling required the deposition of 200 nm amorphous carbon and more than 2 microns Pd layer onto the deposited metal patterns followed by high-energy processing, which introduced heavy weights and stresses to be pressed onto the DNA-templated thin metal layers. Eventually, once the DNA-templated metal pattern could not resist the external stresses, the 3D space-and-line morphology collapsed into the planar morphology, as being observed at 2.5 nm and 2 nm metal thicknesses.

The collapse of the 3D morphology correlated with weak side-connection strength of the DNA-templated metal patterns. At the metal thicknesses of 2.5 nm and 2 nm, because metal thicknesses were not significantly higher than the groove depth (~2 nm), the side connection could barely form. The resulting weak connections, as indicated by the cracks and different contrasts in the metal layers (signs of film discontinuity in Supplementary Fig. 21 d and e), failed to resist the stresses during the FIB milling. As results, only planar morphology could be produced. In contrast, for thicker metal

layers (6 nm and 3.7 nm), the metals growth exceeded the groove depth, and formed strong side connections with those on top of the DNA lines. As indicated in the cross-section TEM images (Supplementary Fig. 21 b and c), continuous ripple-like metal films with intact side connections, were not fully planarized during FIB. Meanwhile, we still observed signatures of the stress-induced distortion. The cross-section shape of DNA lines deviated from the designed rectangular shape into the trapezoid shape, as well as the descent groove depths. Notably, because FIB milling deformed DNA templates, it remained challenging to use the FIB milling for exploring the metal growth dynamics at the DNA sidewalls.

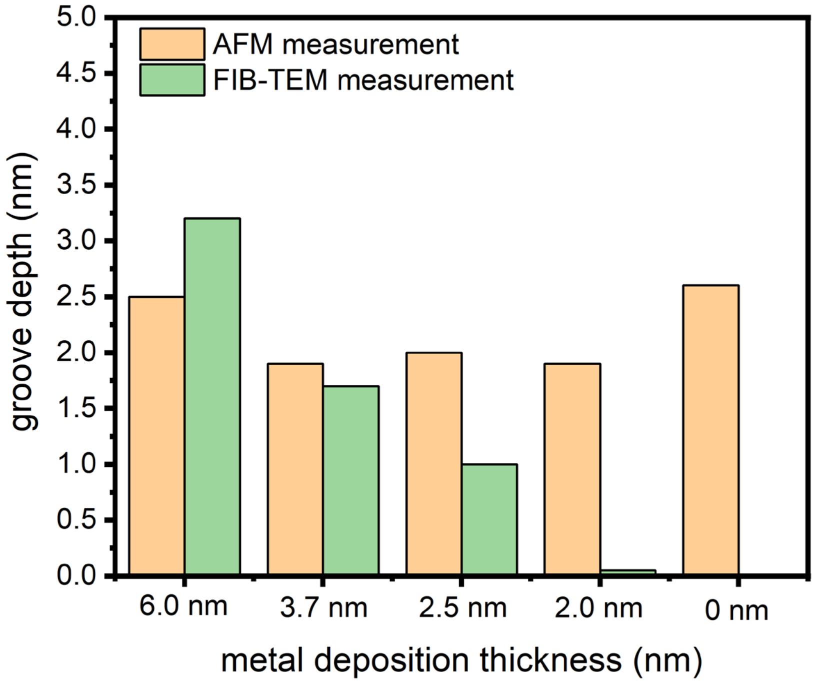

Supplementary Figure 20. The groove depths measured by AFM and TEM at different Pd thicknesses. Notably, we did not perform FIB for DNA templates without metal deposition, because of their fragile nature of DNA structures under high-energy processing.

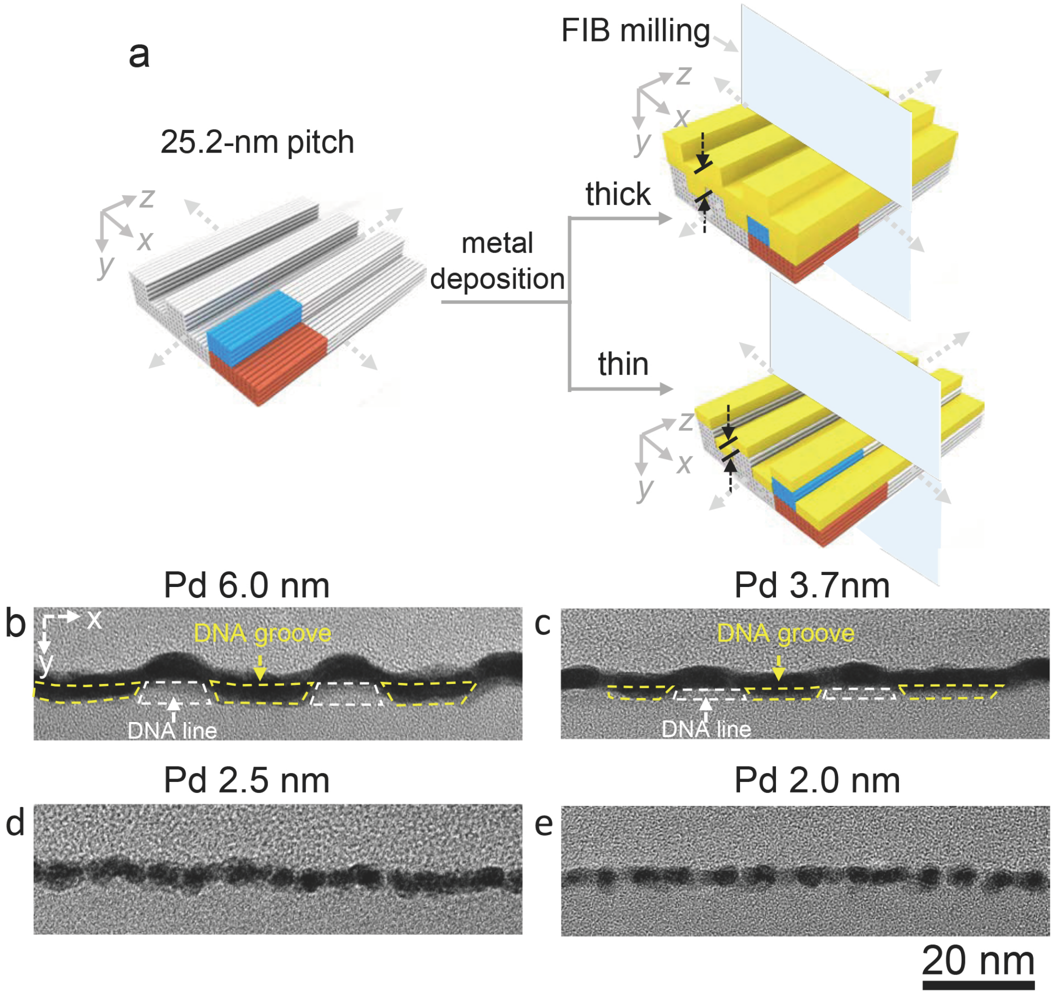

Supplementary Figure 21. FIB-TEM process for DNA templates coated with different Pd thicknesses. (a) Schematic for the deposited Pd of different thicknesses and the subsequent FIB process. (b-e) The cross-section TEM images for 6 nm (b), 3.7 nm (c), 2.5 nm (d) and 2.0 nm (e) Pd thicknesses. The white and the yellow dashed circles indicated the DNA line and the DNA groove regions after metal deposition, respectively.
